# Intelligent high‐throughput intervention testing platform in *Daphnia*

**DOI:** 10.1111/acel.13571

**Published:** 2022-02-23

**Authors:** Yongmin Cho, Rachael A. Jonas‐Closs, Lev Y. Yampolsky, Marc W. Kirschner, Leonid Peshkin

**Affiliations:** ^1^ Department of Systems Biology Harvard Medical School Boston Massachusetts USA; ^2^ Department of Biological Sciences East Tennessee State University Johnson City Tennessee USA

**Keywords:** *Daphnia magna*, intervention testing platform, long‐term culture, machine learning, phenotypic age, phenotyping

## Abstract

We present a novel platform for testing the effects of interventions on the life‐ and healthspan of a short‐lived freshwater organism with complex behavior and physiology—the planktonic crustacean *Daphnia magna*. Within this platform, dozens of complex behavioral features of both routine motion and response to stimuli are continuously quantified over large synchronized cohorts via an automated phenotyping pipeline. We build predictive machine‐learning models calibrated using chronological age and extrapolate onto phenotypic age. We further apply the model to estimate the phenotypic age under pharmacological perturbation. Our platform provides a scalable framework for drug screening and characterization in both life‐long and instant assays as illustrated using a long‐term dose‐response profile of metformin and a short‐term assay of well‐studied substances such as caffeine and alcohol.

## Graphical abstract

We present a high‐throughput scalable platform for intervention testing in *Daphnia*. The platform is used to quantitatively characterize dozens of phenotypic features and to build a predictive model of phenotypic age.

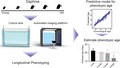

## 1 INTRODUCTION

Developing pharmacological interventions that slow down the aging process and consequently postpone the onset and progression of age‐associated diseases is highly sought after. The aging process can be actively regulated by multiple interventions such as environmental, genetic, and pharmacological factors. Specifically, a large body of research has found that a reduction in caloric intake can extend lifespan and delay disease onset in a wide range of species from nematodes to primates (Clancy et al., 2002; Colman et al., 2009; Fontana et al., 2010; Greer & Brunet, 2009; Mair & Dillin, 2008). A variety of heritable variants have been identified that to some extent mimic caloric restriction. For example, a *Caenorhabditis elegans* mutant for the insulin receptor *daf*‐*2* can live two to three times longer than wild‐type animals (Kenyon et al., 1993), and flies and mice that have mutations in the insulin or insulin‐like growth factor‐1 receptor gene similarly show an enhanced lifespan (Blüher et al., 2003; Holzenberger et al., 2003; Tatar et al., 2001). Drugs that affect hypothesized metabolic pathways responsible for caloric restriction, such as rapamycin, metformin, and resveratrol, are being studied for their potential to enhance lifespan in several organisms from nematodes to mice (Cabreiro et al., 2013; Harrison et al., 2009; Martin‐Montalvo et al., 2013; Mouchiroud et al., 2010). Pharmacological interventions are currently the most practical strategy for affecting aging in humans, avoiding the technical and ethical problems with genetic interventions and the difficulty of maintaining an unpleasant, life‐long calorie‐restricted diet. However, since the mechanisms driving the aging process are not well understood, there currently exist few druggable targets for anti‐aging treatments, and therefore, the evaluation of drug effects on the aging process requires the development of new high‐throughput screening platforms.

A key component of new high‐throughput screening platforms will be identifying quantitative biomarkers of aging. Since individuals may not age at the same rate, quantitative biomarkers of aging are valuable tools to measure phenotypic age, assess the extent of healthy aging, and potentially predict not only health and lifespan but also age‐related outcomes for individuals within a population, even at an early age. Molecular biomarkers (often based on gene expression) are robust quantitative metrics and can reflect some of the molecular mechanisms underlying the aging process (Butler et al., 2004; Xia et al., 2017), but often require sacrifice of the subject, laborious sample processing and constitute a single data endpoint. Phenotypic biomarkers can be harder to quantify but are fairly easy to obtain, non‐invasive, and therefore are possible to repeatedly assay over the entire life of the subject and in future generations. In this regard, walking speed, the “chair stand test,” standing balance, and body mass index are well‐known biomarkers of aging in humans (Xia et al., 2017). While these assays demonstrate the possibility of using phenotypic information as aging biomarkers, for obvious ethical and time considerations humans are an unsuitable model for the initial screening of chemical compounds or other interventions that may ameliorate age‐associated phenotypic declines. Therefore, the development of novel model systems and their aging biomarkers will enable the discovery of potential anti‐aging therapeutic strategies for humans.

The study of aging in a simple, short‐lived model organism is extremely attractive. The small crustacean *Daphnia* (also called “water flea”) promises to be a powerful pharmacological model organism for several reasons: (a) it is a diploid, parthenogenetic species with a relatively short median lifespan (~50–100 days, depending on environmental conditions) (Dudycha, 2003; Dudycha & Tessier, 1999; Kim et al., 2014; Robinson et al., 2012), (b) it has a short reproductive cycle (e.g., a female can produce a clutch of 1–25 neonates every instar [Smirnov, 2017b]), and (c) its genome and complex body plan are significantly more homologous to humans than a common aging model worm *C*. *elegans*, thus allowing more human‐relevant, tissue‐specific manifestations of aging to be analyzed (Ebert, 2005). These properties of *Daphnia* allow short timeline experiments with a large sample size required for aging research, while their complex phenotypes provide opportunities to build strong phenotype‐based biomarkers to assay the effects of drugs on the aging process. One of the most important parameters in drug development is the absorption, distribution, metabolism, excretion, and toxicity (ADME‐Tox) of drugs. *Daphnia* allows the ease of perturbation by small molecules compared to other short‐lived invertebrate model organisms such as *C*. *elegans* and *Drosophila*, which have impermeable cuticles that form strong barriers to the absorption of drugs. *Daphnia* is a common model organism widely used in ecotoxicological testing (Bownik, 2017; Guilhermino et al., 2000): They display high permeability and high sensitivity to compounds in their environment, which is an essential characteristic for drug screening (Flaherty & Dodson, 2005; Guilhermino et al., 2000; Oliveira et al., 2016). Prior studies have demonstrated that the behavior of *Daphnia* is altered by chemicals, nanoparticles, pesticides, or bacteria products (Bownik, 2017), which allows for dose‐dependent tests. In contrast, *C*. *elegans* and *Drosophila* are non‐aquatic species, and therefore, it is difficult to accurately profile dose dependence of pharmacological perturbations because of high individual variation in effective drug consumption. Thus, the short‐lived, freshwater crustacean *Daphnia* offers a number of advantages over other common models of aging for screening of novel pharmacological agents.

Here, we introduce a unifying framework for evaluating the effectiveness of anti‐aging interventions using *Daphnia* as a model organism and a machine‐learning algorithm to build a predictive model with longitudinally tracked phenotypes. Specifically, we designed a scalable culture platform for longitudinal monitoring of *Daphnia*. With this platform, we tracked animals in a cohort of *Daphnia* until their natural deaths by developing a computer vision algorithm that quantitatively extracts a representation of the location and behavioral parameters of individual animals in the culture tanks. The machine‐learning approach provides methodologies for the analysis and practical use of the collected multi‐dimensional phenotypic information, reaching meaningful biological conclusions. The major strength of machine learning is the potential to identify relevant patterns within complex, nonlinear data without the need for any *a priori* mechanistic understanding of the underlying aging processes and to iteratively improve the predictive performance of models. We therefore used extracted features to train a supervised machine‐learning algorithm to predict phenotypic age and compare to chronological age. We found that our predictive model was able to accurately estimate phenotypic age that might reflect animals’ health states. We then evaluated the robustness of our model in experimental conditions such as drug or chemical treatment and examined how much these perturbations affected the animals’ healthspan. The developed analysis pipeline allows for quick and efficient tests for potential pharmacological candidates that increase the healthspan. The high‐throughput, scalable, automated approach presented here enables the extraction of new behavioral features and the training of the model to evaluate the effect of perturbations on behavioral outputs according to experimental purposes.

## 2 RESULTS

### 2.1 A scalable culture and high‐throughput longitudinal phenotyping platform

We designed a platform to enable us to culture *Daphnia* at scale with high‐throughput longitudinal phenotyping capabilities. While behavioral monitoring platforms have been widely developed for model organisms such as mice, fly, and nematode (Churgin et al., 2017; Dankert et al., 2009; Hong et al., 2015; Le et al., 2020; Zhang et al., 2016), these platforms are not suitable for aquatic animals. Several platforms for the behavioral study in either fish or *Daphnia* are available but they do not meet our requirements. Most current *Daphnia* behavior tracking platforms have been designed for short‐term experiments (e.g., a few minutes to hours) with a small number of animals (usually ~5–10 animals per tank) for toxicological tests (Bownik, 2017; Kunze et al., 2016; Simão et al., 2019). Understanding the relationship between phenotypic changes and the aging process requires longitudinal monitoring with a large number of individuals because of the stochastic nature of aging processes.

**FIGURE 1:**
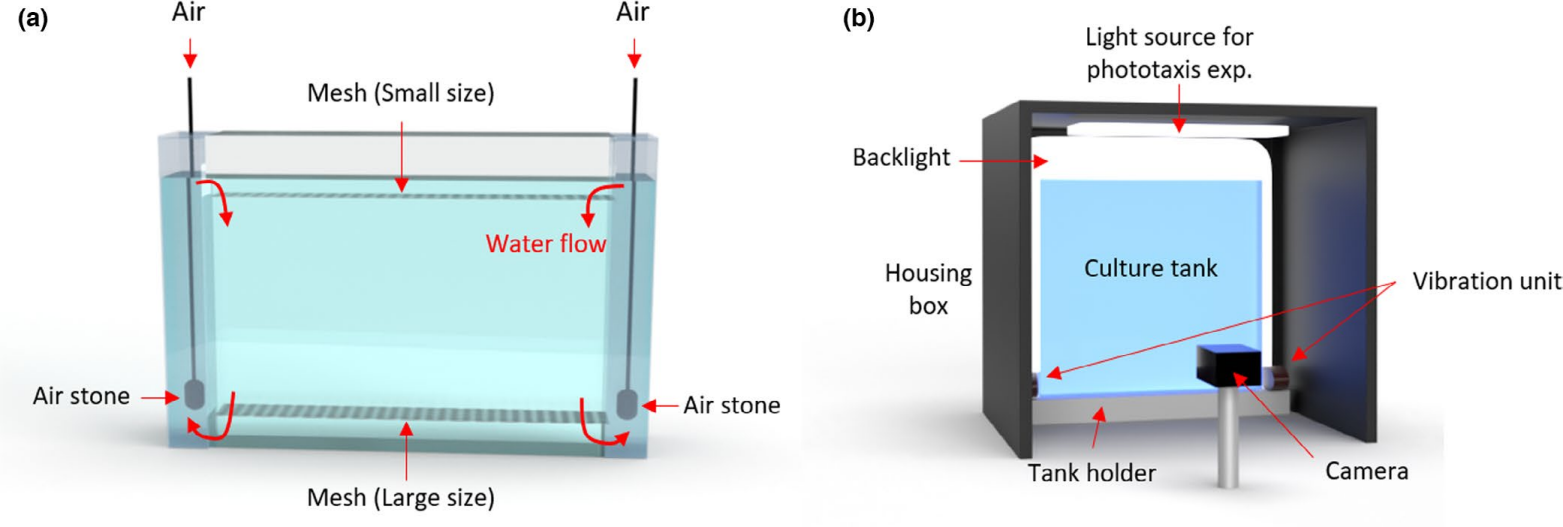
Our platform enables long‐term culture and monitoring of daphnid behaviors. (a,b) Schematic illustrating the customized culture and imaging setup. (a) An individual tank. Air stones, connected to the air source, are located in two side columns to create an aerobic environment for daphnids and to separate neonates from mothers via two different sized meshes. (b) Schematic of imaging setup. A tank is set into the imaging setup and recorded via a frontal camera in a computer‐controlled environment. An even backlight illumination is used constantly. A housing ceiling light is used for the stimulated phototaxis. The scaffolding ensures an invariant tank placement. Four vibrational motors on both sides of the scaffolding are used for the delivery of vibrational stimulus

A further complication in the long‐term culture of *Daphnia* is the need to remove neonates. Since an adult female produces eggs every 3–4 days until death and neonates grow rapidly (Smirnov, 2017c), we should adequately separate neonates from mothers to maintain age‐synchronized cohorts. However, this is still performed manually and is a labor‐intensive and time‐consuming task. To improve upon existing imaging systems for the long‐term and a large number of animals, we engineered an integrated platform that provides (a) a long‐term culture with a large sample size, (b) scalability provided by making an individual culture tank a module, and (c) controllability of stimuli for profiling behavioral responses of daphnids by implementing the mesh‐based tank design and an integrated behavioral assay platform (Figure 1).

To easily separate neonates from mothers and to monitor animals’ phenotypes, we designed a tank which consists of three modules: a housing tank, an insert, and a cap with two different sizes of mesh (850 µm mesh at the bottom of the insert and 300 µm mesh at the bottom of the cap) (Figure S1a,c). To provide sufficient air to the daphnids, we created a continuous air‐lift water flow through the two side columns which are separated from the insert tank where animals are housed (Figure S1a,b). Because of the continuous flow system, neonates are naturally passing through the large mesh at the bottom of the insert tank and get caught at the small mesh of the cap. Therefore, to separate neonates, we just need to remove and wash the cap. When we tested this platform, all 34 neonates were separated from the main tank within 5.5 min (Movie S1). The geometry of the tank is optimized for the 1L volume of media to culture a large number of animals (the size of a housing tank: 23 cm (w) × 20.5 cm (hr) × 4 cm (d)). *Daphnids* can swim freely in a relatively large arena (the size of our insert is 16.5 cm (w) × 14.5 cm (hr) × 2.5 cm (d)). In addition, we can maintain and scale‐up multiple different experimental conditions in a single experimental period (e.g., test different drugs, multiple drug concentrations, or food level at each tank depending on the experimental design and progress) because of the modularity of the system. It also represents truly independent replicates.

Behavioral impairment is one of the noticeable age‐related changes observed across many species (Arey & Murphy, 2017), and so we wanted to include an assessment of this within our platform. Since light and vibration are stimuli well‐known to induce behavioral responses in *Daphnia* (Smirnov, 2017a), it would be essential to accurately control the light and/or vibration stimulus and monitor its responses with age to measure the effect of the perturbation on the behavioral performance. To allow precise temporal and strength control of stimuli and recording under the controlled environment, we developed an automated imaging setup (Figure 1b). All parts (e.g., camera, lighting, vibrational motor, and data organization) are integrated and controlled via an Arduino board and a custom MATLAB GUI, which enables automatic experimental setup, controlling of stimulus intensity and timing, and monitoring of the phenotypic changes of the *Daphnia* as they age (Figures S2 and S3).

### 2.2 A computer vision algorithm for extracting quantitative phenotypes

**FIGURE 2:**
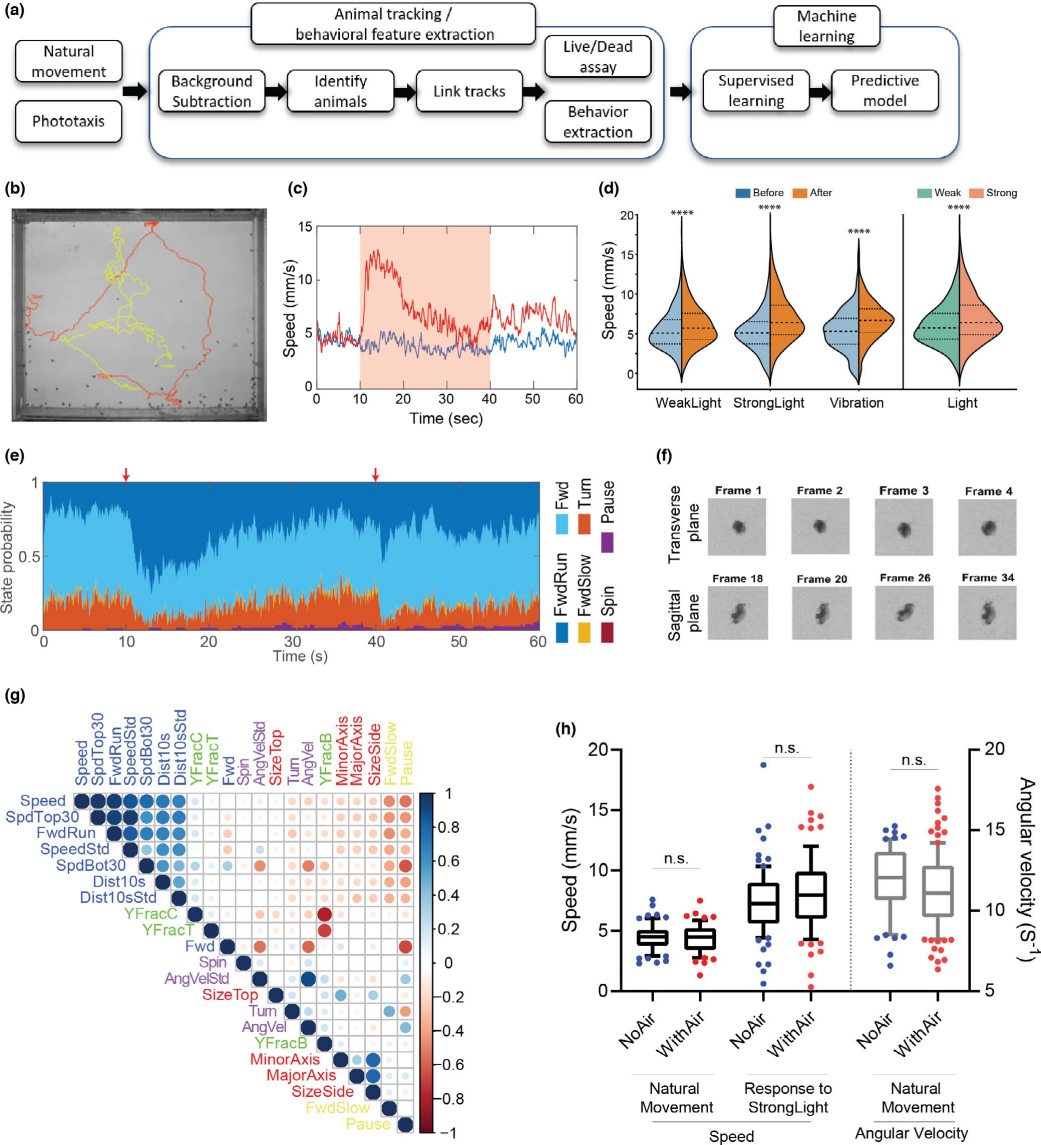
Quantitative behavioral analysis. (a) A workflow of the behavior tracking and predictive model building. (b) Two sample animal trajectories in a phototaxis experiment. (c) Examples of extracted speed in (i) normal and (ii) 30s light‐stimulus conditions. The shaded light red box indicates the timing of a light stimulus. (d) Speed changes in response to various stimuli: weak light, strong light, and vibration (Day 16; *n* = 424). (e) Descriptive behavioral features in 30s light‐stimulus conditions (Red arrows indicate the timing of light on/off (10s to 40s); see Table S1 for details). (f) Example images of the animal for specific size extraction. Using circularity as a criterion, we distinguished the size of the transverse and sagittal planes (See methods for details). (g) The correlation matrix of features, extracted from the natural swimming conditions (Using all longitudinally recorded videos). (h) Comparison of two quantitative features, speed and angular velocity, between no air and continuous airflow conditions with the same animals at the same age (*n* = 67). Statistical analysis: Mann–Whitney test (*<0.05, **<0.01, ***<0.001, ****<0.0001)

To extract multiple behavioral features quantitatively, there is a need for a robust analysis pipeline. Even though there is commercially available software for *Daphnia* behavioral monitoring, it has been applied to a limited number of animals in a tank (~5 animals per tank) for a short period of time (a few minutes to hours) (Simão et al., 2019). To process the data from our high animal‐density experimental conditions, we developed a custom MATLAB script that extracts various behavioral and morphological features to track phenotypic changes during the aging process. Briefly, we performed background subtraction and image segmentation to identify and determine the location of live individuals (Figure 2a, Figure S4, and Note S2). Using a consensus approach informed by positional and morphological parameters, we separated the individual animals from other objects, such as dust or gunk, and performed tracking followed by measurements of the behavioral features (Figure 2b).

When we considered which features can be extracted, we started with parameters that had previously been associated with toxicological testing (Bownik, 2017). For example, speed has been shown as one of the sensitive parameters in many *Daphnia* toxicology tests (Bownik, 2017). The vertical movement in a phototaxis experimental condition can be another useful feature. Therefore, we monitored animals’ movement in two regimes, natural swimming and stimulus‐induced response, to examine age‐related phenotypic changes in both cases. For example, the average swimming speed showed different patterns between these regimes. In the natural swimming mode (i.e., no external stimulus), we observed relatively consistent swimming speed. Yet, when animals were exposed to additional light, they showed an obvious response: The average speed increased dramatically as animals moved up toward the light (Figure 2c). Eventually, the speed stabilized at the level we observed prior to switching the light on. On the contrary, once the light was turned off, animals moved toward the bottom of the tank (Figure 2c,e, Figure S5, and Movie S2). Furthermore, our platform allows controlling the brightness of light (Strong light: 2.65 ± 0.02 kLux and weak light: 0.75 ± 0.01 kLux). Therefore, we tested how daphnids responded to different strengths of light stimuli. Lastly, since vibration is known to evoke daphnids’ responses (Smirnov, 2017a), we monitored the animals’ responses to controlled vibrational stimulus. Figure 2d shows the distribution of speed before and after each stimulus. *Daphnia* clearly responded to each stimulus and showed graded responses to the different strengths of light, which indicates that the controlled stimuli in our platform can induce daphnids’ behavioral responses.

Several studies in various model organisms have shown that morphological parameters such as muscle mass, body weight, and size are associated with lifespan or diseases (Ebert, 1991; Shimada & Mitchison, 2019; Swindell et al., 2008). Thus, we also extracted morphological features related to animals’ size (Figure 2f and Movie S3). Furthermore, we created a list of locomotor classifications such as “forward fast running (FwdRun),” “forward swimming (Fwd),” “forward slow swimming (FwdSlow),” “turning (Turn),” “spinning (Spin),” and “pause” which involve basic locomotor actions of *Daphnia*. The analysis pipeline computed the per‐frame probability of each locomotor description. Figure 2e shows that most of the time, young and healthy animals show forward swimming (either FwdRun or Fwd). The next‐most common behavior was turning, in which *Daphnia* made a large, rapid change in orientation. But when the light stimulus was delivered, most animals changed their behavioral status from either forward swimming or turning to forward fast running. It indicates that our descriptors of behavior are robust in reflecting animals’ behavioral changes. In total, we extracted a set of 21 quantitative features focused on natural swimming behaviors and 12 additional features related to phototactic response (Table S1). To understand the relationship among extracted features, we calculated the correlation matrix (Figure 2g). As expected, features in different categories are largely independent of each other, but ones in the same category (e.g., the forward movement features, turning features, pause mode, morphology‐related features, and vertical location‐related features) are more correlated.

Since we created a continuous flow for 24 hr to create air‐driven circulation in the tank, except when we recorded the video, we tested whether this continuous flow itself alters the animals’ behavior. There were no statistical differences between no‐air and with‐air flow conditions (Figure 2h) in both speed and angular velocity of the daphnids. This suggests that our continuous flow is nonrestrictive enough to not alter daphnids’ swimming patterns. Furthermore, to evaluate the performance of the automated algorithm, we segmented the one minute video into four overlapping segments (e.g., 30 s each: 0–30, 10–40, 20–50, and 30–60s, respectively) and compared the results of processing of each fragment for the features including speed and angular velocity. We found that the replicated data are not significantly different among the fragments (Figure S6). It indicates that our algorithm was able to measure physiological features in a robust, reproducible manner.

### 2.3 Highly controlled survival probability across replicates in the platform

The platform allows one to count the number of live animals for a survival assay, to build a lifespan curve, and ultimately to evaluate the effect of perturbations on lifespan. However, the manual live/dead assay is a heavily labor‐intensive task, and to our knowledge, there is no available algorithm for a *Daphnia* lifespan assay. Therefore, an automated counter is required to build lifespan curves from our large‐scale high‐throughput longitudinal experiments. However, since animals often overlap or touch each other in the high‐density population case, it is not easy to accurately segment and detect all individuals to count them. Since our algorithm could not count all live animals perfectly (e.g., count 75.62% of animals with 0.29% error [mislabeling]), we developed a hybrid “human‐in‐the loop” approach. A MATLAB GUI is used to manually curate the counts (Figure S7). Since more than 75% of animals are already automatically and correctly counted, this pipeline makes counting much faster than an entirely manual approach and much more accurate than an entirely automated one.

**FIGURE 3:**
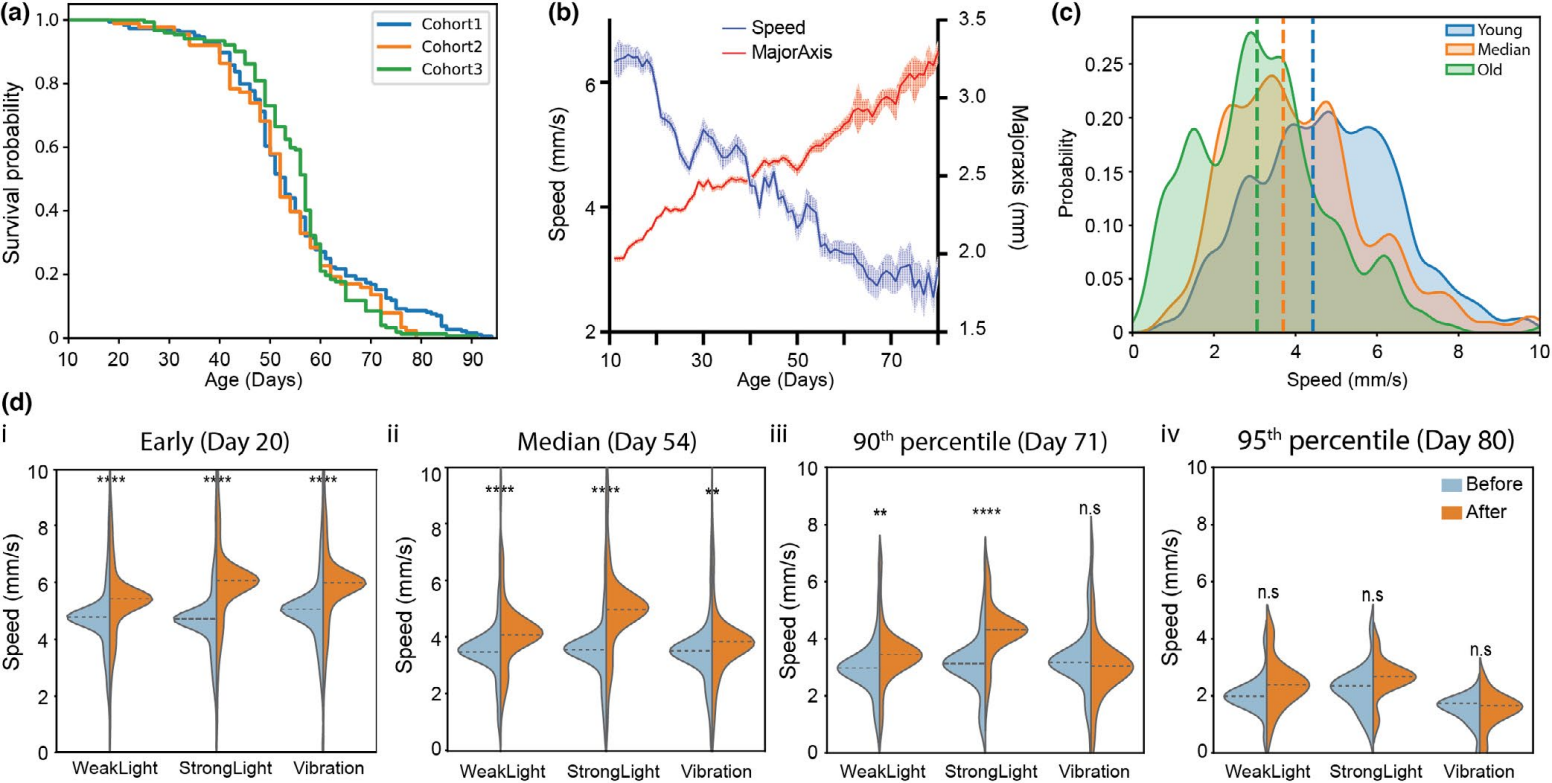
Age‐related phenotypic changes. (a) Lifespan curves for three control experiments (Cohort 1: *n* = 184, Cohort 2: *n* = 88, and Cohort 3: *n* = 152). (b) A lifetime population average of speed (blue) and body size (red) of *daphnids* in a control experiment (*n* = 424). Error bars are SEM. (c) Density plot of speed at three ages: young (blue—20th percentile; *n* = 346), median (red—54 day old; *n* = 217), and mature (green—80th percentile of the lifespan; *n* = 96), (d) Response to stimuli (weak light, strong light, and vibration) speed before (blue) vs after stimulus (orange) at four ages (i: young, *n* = 423, ii: median, *n* = 217, iii: mature, *n* = 56, and iv: old, *n* = 20). Two‐tailed *t* test (** *p*‐value <0.01, **** *p*‐value <0.0001)

Reproducibility in the new platform is one of the critical factors in finding robust age‐related biomarkers and then conducting phenotypic screens for pharmacological interventions that promote healthy aging. We tested whether three independent cohorts could show reproducible lifespan curves. Figure 3a shows that reproducibility between cohort experiments is high (e.g., no statistical difference with the log‐rank test; median lifespan: cohort 1 = Day 53, cohort 2 = Day 52, and cohort 3 = Day 57). Not surprisingly, individual trial reproducibility is relatively low (Figure S8a). It serves to inform us that the general health of the natural variants is well controlled in our platform if the size of the sample is large enough (> 90 animals per cohort). It indicates the feasibility of the platform to test age‐related interventions.

The comparison between survival curves obtained in replicate cohorts in the new platform to those obtained by us using the same *Daphnia magna* clone in comparable conditions but under the standard lifetable protocols (Figure S8b) reveals that the new platform increases cohorts’ lifespan. Furthermore, we conducted the parametric modeling by fitting generalized gamma, Weibull, Gamma, Exponential, Log‐logistic, Log‐normal, and Gompertz models to our data and to several standard protocol studies conducted using the same *Daphnia magna* genotype. The assay reveals that generalized gamma distribution shows the best fit to the data in most cases, followed by Gompertz distribution (standard protocol) or Log‐logistic distribution (our platform) (Figure S8c,d). Lastly, a comparison with median lifespan estimates obtained by different laboratories using different *Daphnia magna* genotypes and implementing a variety of traditional lifetable protocols reveals a remarkable reproducibility of the data generated by our platform (Note S4).

### 2.4 Age‐related phenotypic changes

Age in animals is accompanied by a decline in locomotion and decreased responses to various stimuli, which are direct measures of healthspan (Herndon et al., 2002). We therefore assessed motility and responses to controlled stimuli over the entire adult lifespan of animals. As expected, the average swimming speed decreased with age, while body size increased at the population level (Figure 3b). Behavioral features display variation at the individual level (e.g., the same animal did not show the exact same behavioral performance on the task), but the population‐based average value shows the trend of age‐related changes. It is known that *Daphnia*, like most other crustaceans, continues to grow their entire life (Smirnov, 2017c). As we mentioned, both light and mechanical stimuli can induce *Daphnia* responses. Thus, we set out to quantify this effect using the speed parameter. Figure 3d shows the change of speed before and after the three different stimuli over the lifetime (we selected four timepoints: young age (Day 20), the median of lifespan curve (Day 54), 90th percentile of the lifespan curve (Day 71), and very old age (Day 80)). When animals are young (Figure 3d i and ii), they significantly respond to all three types of stimuli, even though strong light induces faster movement than weak light. When animals get older, the distributions of speed for both before and after stimuli are gradually decreased. Specifically, at age Day 71, animals still clearly respond to both light stimuli, but vibrational stimulation does not induce significant responses (Figure 3d iii). At old age (Day 80), animals do not show significant responses (Figure 3d iv). This establishes that our platform is sufficiently sensitive to capture the change of animals’ behaviors with age.

### 2.5 The effect of metformin treatment on both the lifespan and phenotypic features

We next asked how pharmacological perturbations influence lifespan and behavioral decline during the aging process. While several potential anti‐aging drugs have been suggested in various model organisms, they have not been well tested in *Daphnia*. As a proof of concept of the suitability of the developed model for the measurement of phenotypic age in perturbed conditions, we used one of the well‐known anti‐aging drugs, metformin, in *Daphnia magna*. Metformin is a drug commonly prescribed to treat patients with type 2 diabetes, and several studies suggest that metformin can increase the lifespan of various model organisms (Barzilai et al., 2016; Cabreiro et al., 2013; Martin‐Montalvo et al., 2013), although non‐significant effects of metformin on lifespan have also been observed (DrugAge database (Barardo et al., 2017)).

**FIGURE 4:**
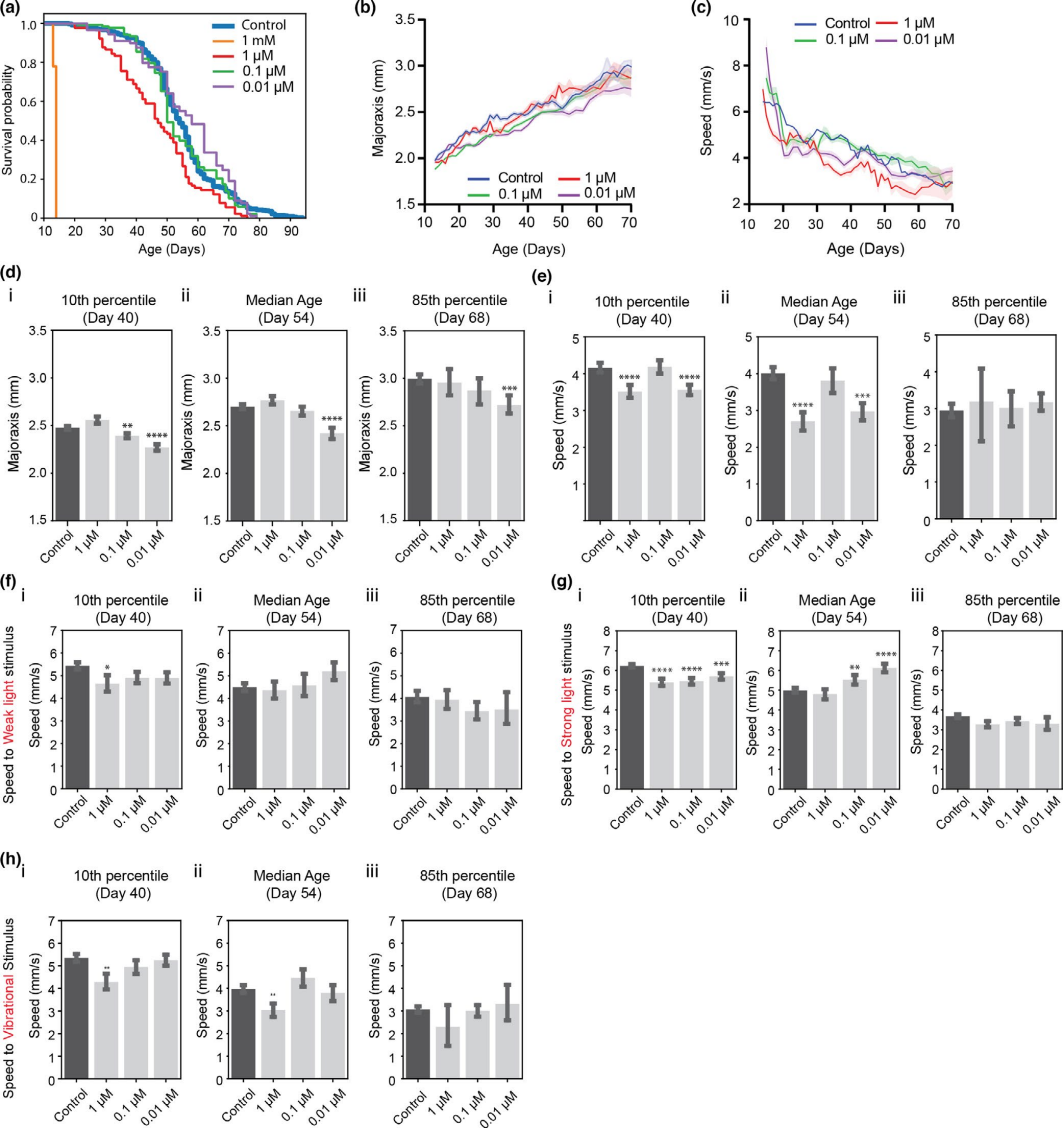
Behavioral changes and longevity in metformin‐treated animals. (a) Survival curves (Control: *n* = 424, Met 1 mM: *n* = 50, Met 1 μM: *n* = 90, Met 0.1 μM: *n* = 138, Met 0.01 μM: *n* = 89), (b) average body size and (c) average speed in the non‐stimulated condition (sample size of each condition is the same as in (a); SEM error bars), (d) Body size and (e) Speed at different ages. (f–h) Response to stimuli (weak and strong light, vibration) at different ages. All tests compared to control via two‐tailed *t* test, *<0.05, **<0.01, ***<0.001, ****<0.0001. (Day 40: *n* = 393, Day 54: *n* = 217, Day 68: *n* = 67)

We first determined the long‐term effects of four doses of metformin in female daphnids (1 mM, 1, 0.1, and 0.01 µM). The age when drug treatment is started may also be an important variable to consider. Since, in mice, early life metformin treatment can extend mean lifespan while late‐life treatment failed to increase lifespan (Anisimov et al., 2011), we started to apply metformin when animals were transferred to our culture platform after they were fully developed (Days 12–13, when they start to produce progenies). Unsurprisingly, we observed a significant decrease in lifespan for daphnids cultured at a high dosage of metformin compared to those at the lower concentrations or the control condition (all animals were dead within 2 days in the toxically high 1 mM metformin concentration), following known trends in other model organisms (Martin‐Montalvo et al., 2013) (Figure 4a). 1 µM metformin is also toxic and significantly shortens the median lifespan of female daphnids by 14.5% (*χ* ^2^ = 20.42 and *p*‐value <0.0001 in log‐rank test). However, the significantly shifted lifespan at the low concentrations of metformin is not observed (Control vs. 0.1 µM: *χ* ^2^ = 1.078 and *p*‐value = 0.297/Control vs. 0.01 µM: *χ* ^2^ = 2.092 and *p*‐value = 0.148 in log‐rank test).

To investigate whether behaviors and morphological features are impacted by metformin treatment, we first plot the changes in body size and speed against age with the result of the control group (Figure 4b,c). Interestingly, the body size of animals treated with 0.01 µM is smaller than the control one over almost the entire lifespan (Figure 4d). In mice, metformin‐treated males weighed less than control animals (Martin‐Montalvo et al., 2013). In 1 and 0.01 µM, the movement in the non‐stimulated condition is slower than in control animals until the median age (Figure 4e). Based on the lifespan assay, 1 µM dose is toxic, likely causing the slowing of swimming speed. 1 µM metformin‐treated animals also show reduced responses to external stimuli (Figure 4f–h). On the contrary, the reduced speed of 0.01 µM‐treated animals might be caused by small body size. In a steady environment, smaller animals move slower, but when responding to stimuli they might be able to react more actively because of the effect of the drug (Figure 4f–h).

### 2.6 Quantitative behavioral metrics can estimate phenotypic ages using machine learning

We sought to determine the degree to which individual features correlate with chronological age. We performed a simple linear regression on each feature for the measured age and evaluated its performance. The major axis parameter shows the best correlation with age, followed by sagittal plane body size (Table S2). While several single parameters were somewhat correlated with chronological age, we can expect that we could build a more accurate predictive model using combined extracted features as input.

**FIGURE 5:**
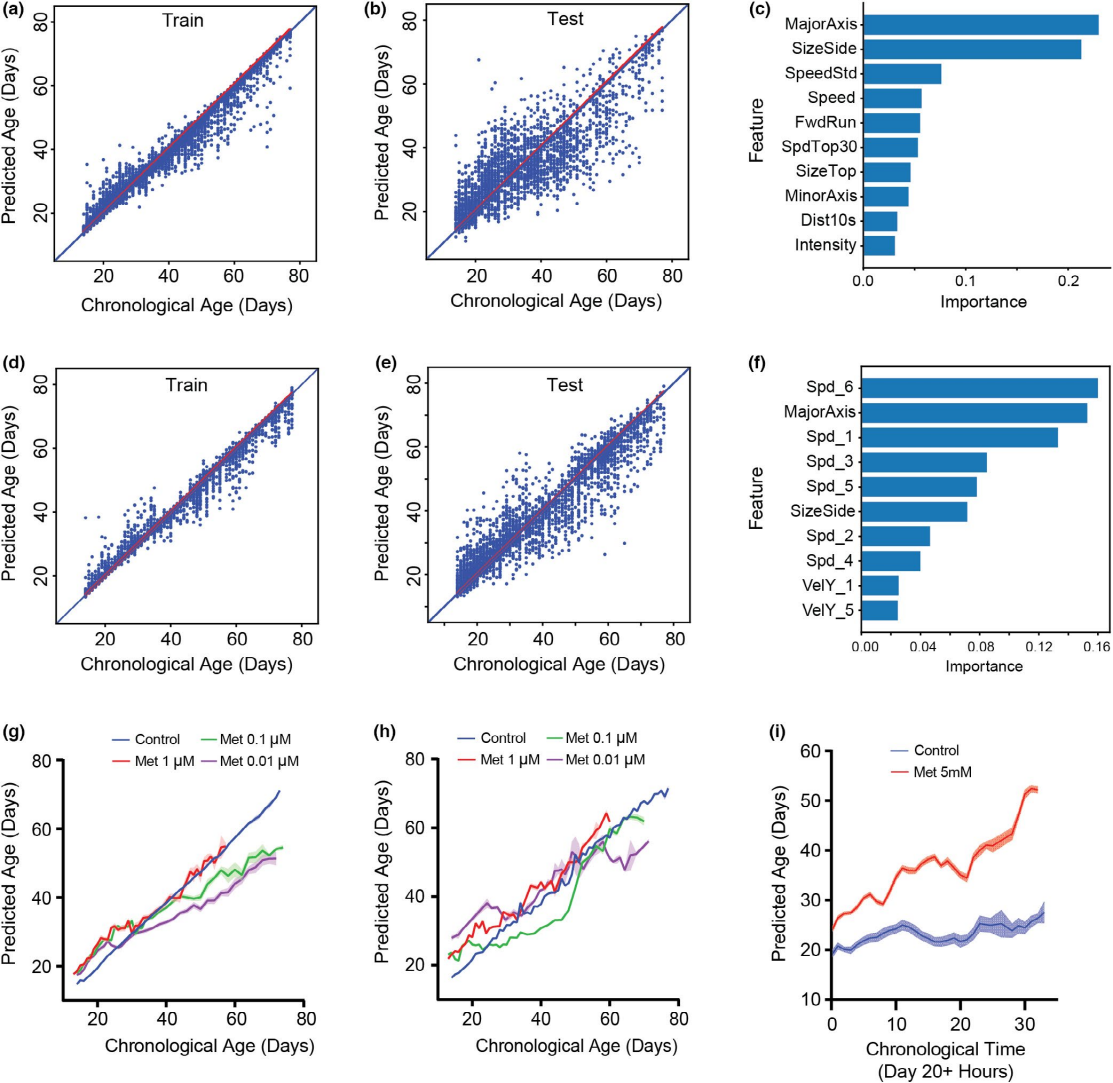
Predictive model using Gradient Boost. (a–f) The model built by Gradient Boosting predicts ages from (a, d) training and (b, e) testing data sets (a, b) the natural (12k individual trajectories) and (d, e) stimulus‐induced condition (16k individual trajectories). The diagonal blue line indicates a theoretically perfect prediction. (See Table S3 for the accuracy test). (c, f) Importance of the top 10 features derived by Gradient Boosting for (c) the natural and (f) stimulus‐induced condition (See Table S3 for the definition of features). (g, h) Comparing predicted age vs chronological age for control and metformin‐treated animals using the model developed by the natural condition (g: natural and h: stimulus‐induced condition). Error bars are SEM. (i) Comparing predicted age vs. chronological age (Day 20) for control and 5mM metformin‐treated animals in every hour recorded stimulus‐induced phenotypes (control: *n* = 22 and 5 mM: *n* = 19)

With the ability to extract multiple quantitative behavioral features, we asked whether these could be compiled into a single predictive model to estimate the phenotypic ages of individuals using the integration of phenotypic parameters as input features and chronological age as output labels. We built models using five types of machine‐learning models—LASSO (least absolute shrinkage and selection operator) (Tibshirani, 1996), Elastic Net, Random Forest (Breiman, 2001), Gradient Boosting (Friedman, 2001), and SVM (Support Vector Machine) (Cortes & Vapnik, 1995)—and quantified the extent to which the model fitted the data in both natural (i.e., no external stimulus) and stimuli‐induced conditions (Table S3). As expected, the multivariate models show better predictive performance than the univariate one, with lower error and a higher r‐squared value. Particularly, the Gradient Boost model had the best accuracy with the highest r‐squared value and lower mean error than other models for both natural swimming and stimulus‐induced experimental data (Figure 5a,b and Figure S9). A decision tree‐based random forest approach also shows very similar performance as Gradient Boost. This could be because tree‐based ensemble models such as Random Forest and gradient boosting can represent complex interactions among features, which linear regressions, such as LASSO and Elastic Net, cannot do (Breiman, 2001). Unsurprisingly, the model built using stimulus‐induced phenotypic features shows better predictive performance than the one using natural swimming conditions. This can be attributed to the stimulus‐induced behavior being more reflective of age‐related performance, and possibly to the fact that we use more features to represent the stimulus‐induced swimming dataset (Figure 5d,e and Table S3).

One of the benefits of the gradient boost method, in addition to a highly predictive model, is that we can rank relevant features using the permutation feature importance analysis, that is, by calculating the incremental error resulting from the feature being excluded from the model (Figure 5c,f). As the result of a simple linear regression for the individual feature shown, size‐related features were identified as the important features, followed by the speed feature. Interestingly, the lists of important features of the models are different based on the experimental conditions (natural vs. stimulus swimming). Specifically, features indicating the responsiveness to the stimulus (speed or y‐directional velocity) come out as important in the stimulus swimming compared to natural swimming. It might imply that parameters showing behavioral performance are highly related to the health status of animals.

The predictive accuracy of our established model suggests that it may be useful for evaluating the lifespan effects of multiple interventions in *Daphnia* many days before their death. Specifically, we hypothesized that the difference between predicted age and chronological age might be representative of phenotypic age, where healthier animals would be estimated to be younger than their chronological age. To test this idea, we calculated this difference in metformin‐treated animals using the predictive models (e.g., using both models built by normal condition data and stimulus condition data) (Figure 5g,h). Both models estimated the age of low (0.1 and 0.01 µM) metformin concentration‐treated animals to be smaller than that of control animals. On the contrary, 1 µM metformin‐treated animals’ predicted ages are usually higher than the control one. If we calculated the slope of the simple linear regression (forced x‐ and y‐intercepts are zero), the control group shows almost one (0.9639 ± 0.0005 by natural condition and 0.9833 ± 0.0007 by stimulus condition) (Table S4). However, the low concentrations of metformin treatments show smaller slope values than the control one. On the contrary, a high concentration of metformin shows slope values larger than 1, which indicates that the rate of the aging process is differently estimated in our model. With the important features for the models, we can assume that lower behavioral activity in 1 µM contributes to the old phenotypic ages by both models (e.g., no significant size difference between control and 1 µM condition [Figure 4d]). In the lower concentrations, both smaller body sizes and behavioral responses may contribute to the younger phenotypic age in both cases. It demonstrated that we can use the predictive model to estimate animals’ phenotypic age.

Furthermore, this model allows us to quantitatively evaluate the toxicity of drugs at an early time point. Figure 5i shows the phenotypic age of both control and 5 mM metformin‐treated animals. In this experiment, we monitored animals’ phenotypes every hour in both groups in the stimulated motion conditions and then estimated the phenotypic age. All animals in the 5 mM treated group died within 1.5 days. Even a few hours later, the predicted age of 5 mM metformin‐treated animals is higher than the control one. Then, the difference of estimated age is getting larger. It opens up the possibility that our model may be used to test the toxicity of drugs on animals’ health within a few hours.

### 2.7 Estimated phenotypic ages in various perturbed conditions

**FIGURE 6:**
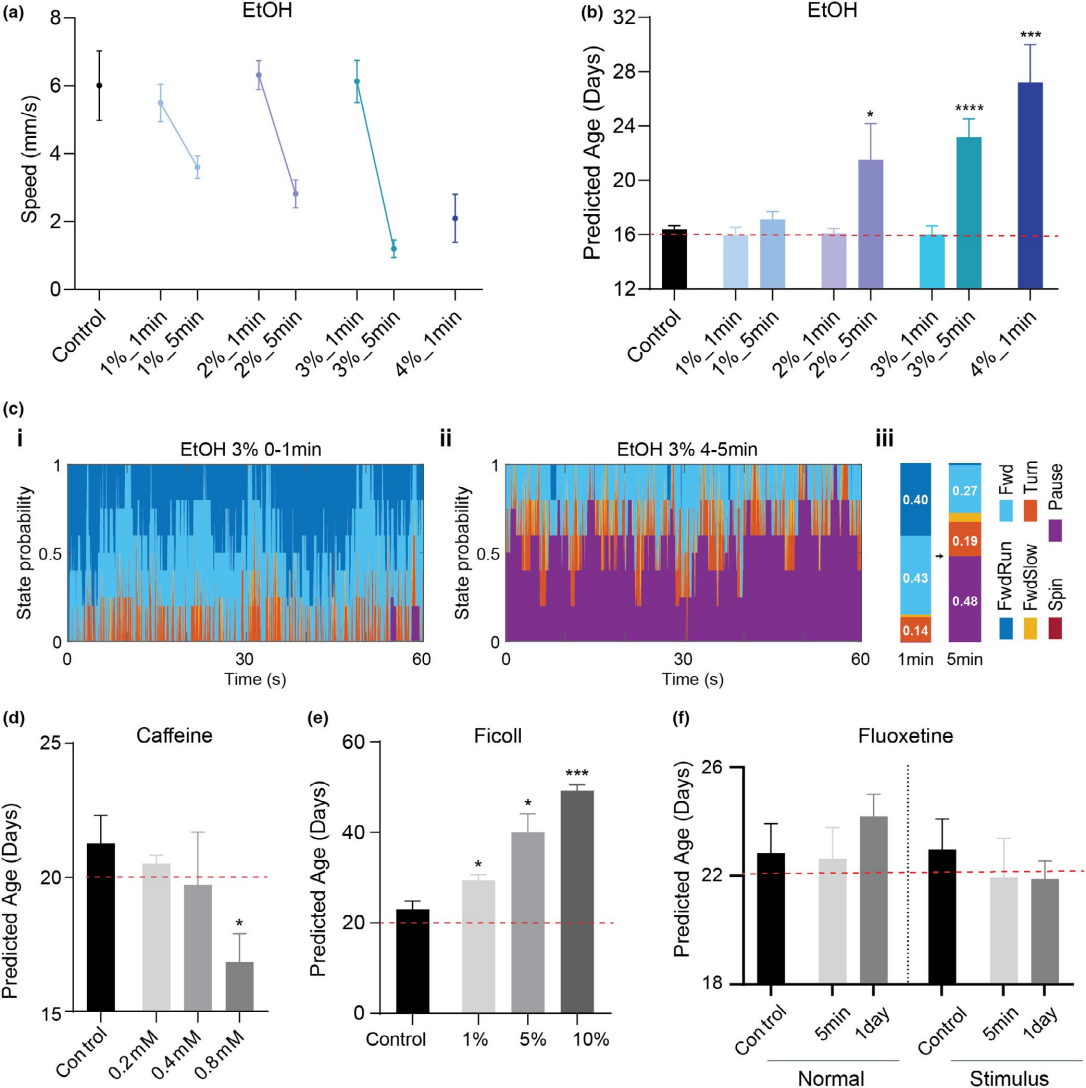
Estimated phenotypic ages in various interventions in young adult daphnids. The red dash line indicates the chronological age. Mann–Whitney test (*p*‐value: * <0.05; ** <0.01, and *** <0.001). Error bars are SEM. (a–c) The effect of ethanol treatment (*n* = 5 for each condition). (a) Speed changes on ethanol, (b) Predicted (Phenotypic) age. The chronological age of the tested animals is indicated by the red dash line. (c) The probability of descriptive behavioral features after 3% ethanol treatment (i: 0–1 min, ii: 4–5 min, and iii: summary). (d) caffeine (*n* = 3 for each condition), (e) Ficoll (*n* = 5 for each condition), and (f) Fluoxetine (*n* = 11)

The key application of our predictive model is to quantify the health of individuals at an early age to test the effects of interventions that perturb animals’ lifespan and healthspan. This avoids the need for monitoring phenotypic changes over the entire lifespan to evaluate the efficacy of drugs. To validate this idea, we tested several chemicals and quantified their using our model. Various studies have reported the effect of chemicals on *Daphnia* physiology, such as movement or heart rate in a short time period. Among them, ethanol was reported to reduce the heart rate of *Daphnia* (Corotto et al., 2010). When we treated daphnids with 1%–4% ethanol, the behavior changed dramatically within minutes (Figure 6a–c). For example, within 5 min of 3% ethanol treatment, animals’ speed sharply decreased (Figure 6c and Video S4). When phenotypic age was calculated for these ethanol‐treated animals, they appeared to be older than the control animals (e.g., control: 16.29 ± 0.89 days, 3% EtOH 5min: 18.05 ± 0.57 days, *p*‐value <0.001). On the contrary, caffeine was reported to increase the heart rate of *Daphnia* (Corotto et al., 2010; Kundu & Singh, 2018). Interestingly, our model indicates that the 0.8mM caffeine‐treated animals appear younger than the controls, which is statistically significant (*p*‐value <0.01) (Figure 6d). To further evaluate our model, we tested solutions of Ficoll, which makes higher viscosity of culture media. Since aquatic animals swim slowly in high viscous media, it is expected that animals’ behavior in Ficoll‐mixed media is inhibited compared to standard ADaM culture. Naturally, our model estimated the much older phenotypic ages for these animals than the control animals (Figure 6e) and reflected the progressive change with the concentration of Ficoll. Lastly, we tested fluoxetine which has been reported to make a slight change in phototactic responses on *Daphnia* (Simão et al., 2019). However, in our analysis, even though the phenotypic age in the phototaxis case is estimated older than the control one, it did not show any significant changes in both natural and phototaxis conditions (Figure 6f).

Furthermore, we used smaller sizes of the tank for the short‐term chemical tests to examine the possibility of reducing the quantity of chemicals and drugs required and the overall cost of the screen. Specifically, we used the original size of the tank (1L volume) for fluoxetine, a medium size of the tank (400ml volume) for ethanol and Ficoll, and the small size of the tank (100ml volume) for caffeine (Figure S9). In all cases, the estimated ages for control are very similar to their chronological ages (Figure 6b,d–f). It indicates that our framework is not specific for a particular design of the experiment. It can be more widely applicable for various shapes of culture tanks depending on the experimental purposes. In conclusion, these test results indicate that our model can predict how old individual animals appear to be and it is sensitive enough to distinguish the effect of various interventions on the health of animals.

## 3 DISCUSSION

Our new platform for lifespan experiments with *Daphnia* cohorts allows scalable, low maintenance, semi‐automatic measurements of lifespan and age‐related changes in this emerging model organism. The platform has a unique environment, which differs from traditional lifetable assays. While traditional assays culture animals in a static water condition, in our platform animals are grown in air‐driven circulation with a mesh bottom (i.e., animals are not in contact with food or any byproduct accumulating at the bottom). Furthermore, while traditional assays involve manual manipulation of animals for water changes and counting, no such manipulation (mechanical disturbance) is required in our platform. It is currently impossible to establish precise causes of substantial lifespan differences across culture condition (Lucanic et al., 2017). What is clear is that there is much less room for human error in handling and substantial gain in scalability (See Note S5) which leads to highly reproducible lifespan curves—a necessary condition for confident evaluation of lifespan interventions.

In addition to lifespan measurements, it is desirable to have quantitative phenotypic biomarkers for the aging process which allow for measuring the health status of animals. This would be essential in the development of new anti‐aging pharmacological strategies. The discovery of such pharmacological interventions in aging requires high‐throughput screening strategies. However, the majority of screens performed in model organisms so far are rather low throughputs with only live/dead assays. Given the complexity of the aging process, the aging biomarkers would be multifaceted, not a single feature. Therefore, measuring various features longitudinally in a high‐throughput manner is a key to establish the meaningful metrics of biomarkers for the aging process and successfully transfer pharmacological approaches to the clinic. For *Daphnia*, although several systems for monitoring behaviors exist, most of them are dedicated to measuring 1–2 features in a short period of time. In this study, we demonstrated an integrated platform that allows for the longitudinal culturing and monitoring of *Daphnia* phenotypic changes with minimal experimenters’ efforts in a high‐throughput manner. The platform's inherent modularity can accommodate various experimental conditions at the same time. With our customized software, we can extract a variety of behavioral and morphological features systematically. Thus, our automated platform and analysis pipeline provides high‐content phenotypic measurements of *Daphnia*.

We set out to perturb *Daphnia* pharmacologically with a known anti‐aging drug and see if we could observe phenotypic changes using our platform. We monitored age‐related phenotypic changes and lifespan in control and metformin‐treated animals until their natural deaths. We found that *Daphnia* shows a shift in its lifespan curves depending on metformin dosage, suggesting that *Daphnia* is sensitive in a dose‐dependent manner to drugs. We also observed that various behavioral features declined during the aging process, as in other model organisms (Le et al., 2020; Martin‐Montalvo et al., 2013). This observation indicates that our platform and pipeline can establish the areas in which *Daphnia* can complement existing models through improvements in sensitivity, cost, or efficiency for aging research and phenotypic screening for anti‐aging drug discovery.

Having established that *Daphnia* lifespan is sensitive to pharmacological perturbation, we next attempted to build a model of the phenotypic clock from an ensemble of quantitatively extracted features. We demonstrated the feasibility of this clock by examining phenotypic changes across different chemical perturbations on a short‐term scale (a few minutes to 1 day). Then, the use of our predictive model can quickly evaluate the effect of these chemicals on the aging process. It suggested that the integration of phenotypic features enables the construction of an estimator for phenotypic ages to explore the relationship between perturbations and the aging process. Note that while such phenotypic traits as velocity and body size change with age, they should not be always interpreted as biomarkers of lifespan or healthspan. A drug that causes animals to appear younger that way might reduce the lifespan or harm animals’ health. Thus being “younger” in terms of modeled phenotypic age only implies a reference to respective biological age in control populations, rather than an assertion of a longer healthier life.

Notably, the performance of the predictive model can be improved. First of all, we can improve the accuracy of the tracking algorithm using convolutional neural networks instead of the conventional image process technique that we used here. Because of the overlapping of animals in a high‐density population, our current algorithm could not track all animals properly during entire recorded frames. A convolutional neural network may provide a better solution for object segmentation and tracking problem. Second, the predictive accuracy could be improved by adding more sample numbers, especially at the older ages, because we have a smaller number of trajectories in old ages compared to the younger ages. Lastly, the model could get an advantage from the incorporation of additional input features. Specifically, by measuring performance on additional behavioral tasks in our platform, such as chemosensation in *Daphnia* (Smirnov, 2017a), we would deeply understand the functional correlates of behavioral changes with normal aging and predict future behavioral impairment.

The major limitation of phenotypic‐based screens is that the mechanism of action of drugs is unknown and there is a possibility to have false positives due to compounds that target mechanisms that could affect the assayed phenotype but are not specific enough to be used as drugs. This limitation would be overcome in combination with innovative approaches to genome, transcriptome, proteome, fluorescent markers, or molecular reporters. By adding multifaceted information, we can investigate integrative biology at high resolution across multiple organ systems, cellular, and genome levels at multiple time points during the aging process. Specifically, the transparent body of *Daphnia* makes real‐time observation of its cell biology and physiology straightforward. With this observation, we can understand how particular organs or systems fail with age. For example, we observed the decreased swimming ability with age that might indicate the degradation of muscle and the decreased performance in phototaxis with age that might indicate the defects in cognitive ability. By measuring muscle loss and detecting damage in neuron number, synaptic integrity, and neurotransmitter, we will find the causality factor for the age‐related phenotypic decline. Thus, using those high‐dimensional multifaceted data to construct dynamic networks that will provide a better understanding of what extent individuals’ age differently and enable assessment of potential interventions by providing a more information‐rich readout.

Using a short‐lived model organism, an automated video monitoring platform and phenotypic profiling pipeline, and machine‐learning algorithms, we show the possibility of that the non‐invasive phenotypic measures could be used in perturbational studies to understand whether a perturbed condition is effective to delay aging at an earlier than its death. In theory, our approach could be adapted to predict phenotypic age for other model organisms. The ability to predict the health status of animals enables us to conduct rapid screens for anti‐aging drugs. We envision that our framework can greatly expand the repertoire of not only high‐throughput behavioral measurements but also deep pharmacological profiling in *Daphnia*. As future work, it is capable of high‐throughput screening large libraries of small molecules for their effects on particular traits of interest, such as extension of healthspan and/or lifespan. Specifically, we can re‐examine drugs from the “DrugAge” database, which compiles results on lifespan effects from >500 distinct compounds in more than 20 species (Barardo et al., 2017). Since very few drugs are tested across multiple species, having most of these compounds profiled in the same organisms during the aging process will create the benchmark for the development of pharmacological strategies that extend the period of healthy life and eventually prevent or reduce the onset of age‐related phenotypic changes or diseases.

## 4 METHODS

### 4.1 Population culture

*Daphnia magna* animals were used in all assays. An IL‐MI‐8 heat‐tolerant clone was obtained from the Ebert laboratory at the University of Basel, Switzerland stock collection originating from a pond in Jerusalem, Israel. To collect synchronized cohorts, neonates born within 1–2 days were separated from the mothers and their sex was determined at Days 8–10. We used a large range of maternal age from Day 11 to Day 90. All mothers are also well‐fed and cultured at the same conditions (25°C incubator). We used females for all experiments in this study to avoid sex‐dependent differences. Collected females were cultured until when animals start to give birth to neonates (approximately Days 9–11 at 25°C), and then, 40–50 animals were randomly assigned to one of our developed culture tanks. All cultures (e.g., mother, neonates, and tank cultures) were maintained in ADaM water (Klüttgen et al., 1994) at the 25°C incubator, exposed to a light cycle of 16 light hours followed by 8 dark hours, and fed every other day the suspension of the green alga, *Scenedesmus obliquus*, at a concentration of 1×10^5^ cells/ml (for 1 animal/20ml density, amount of food prorated by population). Every fourth day, the water was changed and offspring were removed manually until animals were transferred to the culture platform. The airflow in the tank was regulated by a pressure gauge. The operating range of pressure was 1–1.5 psi depending on the number of connected tanks.

### 4.2 Imaging tank fabrication

A custom‐designed transparent tank for *Daphnia* was made from acrylic sheets (McMaster‐Carr, USA). A single tank setup consists of three pieces: a housing tank, an insert, and a cap (Figure S1). Based on the dimension of the tanks, acrylic sheets were cut using a laser cutter. The cut pieces were bonded by acrylic cement (SCIGRIP 16, USA). The housing tank is partitioned into three parts as follows: two side columns were used for air generation, and the middle part for the insert was used for recording. The bottom of the insert was made from an 850 µm mesh to separate progeny from their mothers. The bottom of the cap was made from a 300 µm mesh to prevent the progeny from getting into the insert.

### 4.3 Video acquisition and stimuli control

The recording was made at a rate of 25 fps using a 1.3 Megapixel monochrome CMOS camera (DCC3240M, Thorlabs) coupled with an optical lens (MVL8M23, Thorlabs). For the longitudinal tracking data, the video was recorded every day until all animals died. A white LED background light (LightPad 930, ArtoGraph) was provided to create an even illumination into the entire tank and a white LED strip was used for light stimulus. Using the neutral density filter, we minimize the intensity of the backlight (0.16 ± 0.01 kLux). To record the video, we moved the tank to the imaging setup and turned off airflow to create static conditions. The animals were then left to acclimate for 2–3 min with the backlight before the actual recording began. We filmed two videos: (a) 1 min video to capture natural swimming behavior and (c) 2 min video to monitor behavioral responses to controlled stimuli (weak light stimulus: 20–30 s; strong light stimulus: 40–50 s; and vibrational stimulus: 70–80 s). All videos were stored using unique file names identifying the cohort, recording time and the experimental condition. The following two recording regimes were used: (a) natural swimming with no extra light stimulus and (b) stimuli‐induced swimming (each stimulus was delivered for 10s and used 10 or 20s as the interstimulus interval). The intensity of strong light stimulus at the top of the tank was 2.65 ± 0.02 kLux and the one at the bottom of the tank was 0.32 ± 0.01 kLux. The intensity of weak light stimulus at the top of the tank was 0.75 ± 0.01 kLux and one at the bottom of the tank was 0.10 ± 0.01 kLux. The strong light stimulus is approximately three times brighter than the weak light stimulus. The tank was first covered by a dark housing box (Figure 1c) to control the amount of stimulus light accurately by minimizing the effect of ambient light and also to induce animals to assemble at the bottom of the tank as the baseline before the light stimulus.

### 4.4 Animal detection and tracking

We performed background subtraction and image segmentation to determine the location and identity of animals. Specifically, the background was calculated as the average of every 25th frame (25 fps) and subtracted from each frame to remove all stationary objects (e.g., corpses and dust). We then segmented moving objects to determine the potential boundary of the animals, and the identities of the animals were determined by a consensus informed by positional and morphological parameters. All segmented animals in each frame were parameterized by the centroid position, size, and major‐ and minor‐axis length. The two body size features of *Daphnia* (e.g., body size of transverse vs. sagittal plane) were determined by the circularity of segmented *Daphnia*: ${\text{Circularity}}_{\text{object}}=4\pi A/P^{2}$, where A is the area and P is the perimeter. Here, the size of the transverse plane was classified when the normalized circularity value was larger than 0.92 and the size of the sagittal plane was classified when the normalized circularity value was smaller than 0.58. These thresholds were empirically selected. If the animals were tracked for less than 10 s, we did not consider it a trajectory (it is possible that a single animal can produce multiple trajectories). The moving average method (using a 3 timepoint window) was used for the time series data (Figures 3b, 4b,c and 5g–i). Although the control of lifespan assay was recorded almost every day (at old ages, we recorded videos every other day), the metformin treatment conditions were usually recorded every other day. Thus, we used ±1 day time window for Figure 3d iv and 4d–h, if necessary.

The code is available in GitHub. (Platform control GUI: https://github.com/dydals320/ImagingGUI). Behavior tracking and analysis code: https://github.com/dydals320/DaphniaBehAnalysis.

### 4.5 Predictive model

Longitudinal tracking data are intrinsically imbalanced throughout lifespan because of the death of animals. To overcome this limitation for the regression performance, we used a minority oversampling technique, SMOGN (Synthetic Minority Over‐Sampling Technique for Regression with Gaussian Noise) (Branco et al., 2017). We tested several machine‐learning models such as LASSO, Elastic Net, Random Forest, Gradient Boost, and SVM in the *scikit*‐*learn* module of python (Pedregosa et al., 2011). The performance of the models was determined with the adjusted *R* ^2^ and RMSE values. The training and testing datasets were split using *StratifiedKFold* (using fivefold cross‐validation in this study) to ensure each fold is a good representation of the whole data. There were 12,356 total data points (individual trajectories) for the normal behavioral condition and 16,517 data points for the stimulus condition in the control group. Missing data were replaced by the mean value for a given feature for a given age group.

### 4.6 Statistics and reproducibility

Samples were randomized and treated under the same condition. The sample sizes were not predetermined with a statistical method. Samples were allocated to groups randomly. Data collection and analysis were not performed blindly. No data were excluded from the analysis. Quantification and statistical parameters are indicated in the figure legends or directly marked in the figure, including the statistical method, error bars, *n* numbers, and *p* values. We applied Student's *t* test, Mann–Whitney test, and log‐rank test to determine statistical significance. Specifically, data in Figures 2d,h and 6 were not normally distributed (using the D’Agostino&Pearson normality test); thus, a Mann–Whitney test was used for the analysis. A Student's *t* test was used for other normally distributed data sets. *p* values less than 0.05 are considered statistically significant. Statistical analyses were performed using Graphpad Prism 9 and Python *scipy* (1.6.0) library. Lifespan distribution fitting was conducted using flexsurv package in R (Jackson, 2016).

## CONFLICT OF INTEREST

The authors declare no conflict of interest.

## AUTHORS CONTRIBUTIONS

All authors conceptualized and designed the study. Y.C. and R.J performed the experiments. Y.C. and L.P analyzed the data and wrote the manuscript. R.J., L.Y., and M.W.K reviewed the manuscript.

## Supporting information

Video S1

Video S2

Video S3

Video S4

Supplementary Material

## Data Availability

The data are available in the supplementary material of this article.
