# Supplementary material for: Intelligent high‐throughput intervention testing platform in Daphnia
Source: Aging Cell. 2022 Feb 23;21(3):e13571. doi: 10.1111/acel.13571 (PMC8920439; doi:10.1111/acel.13571)
Supplement: Supplementary file 5 — Supplementary Material [file ACEL-21-e13571-s004.docx]

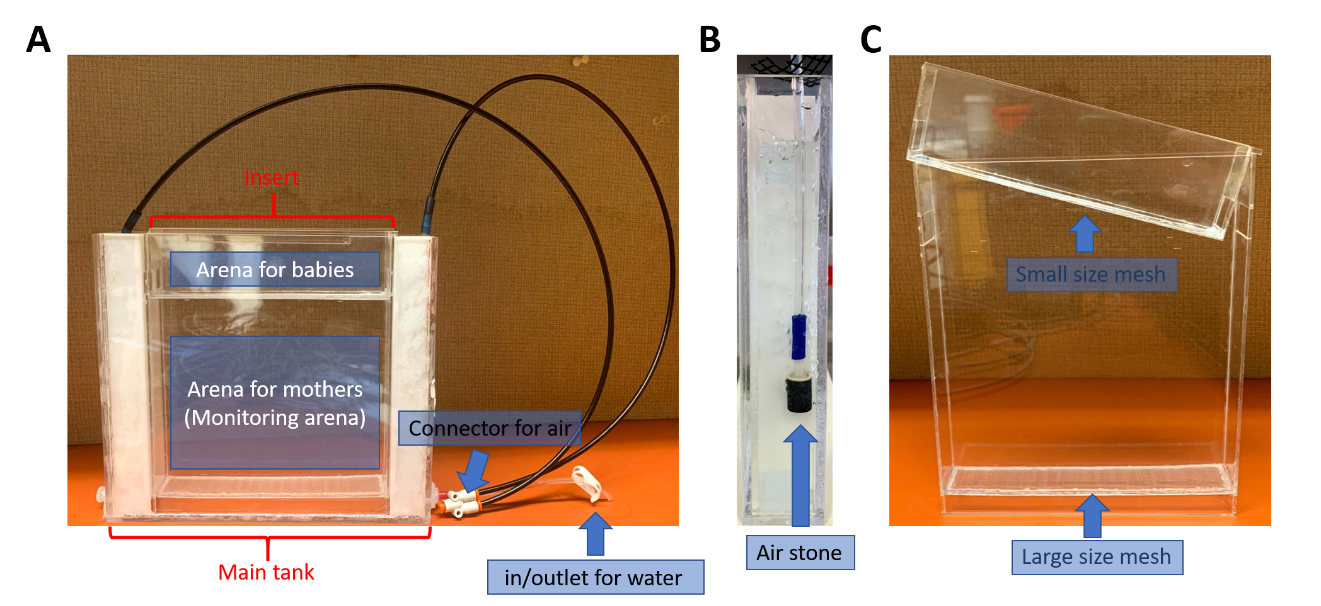


**Supplementary Figure 1.** Images of the tank module.

Picture of an actual tank, consisting of three parts: 1) (A) Main tank has (B) two side columns in order to create air-bubble, 2) (C) the insert has large mesh at the bottom to separate progenies from mothers, and 3) the cap which allows for capturing separated progeny using a fine mesh.


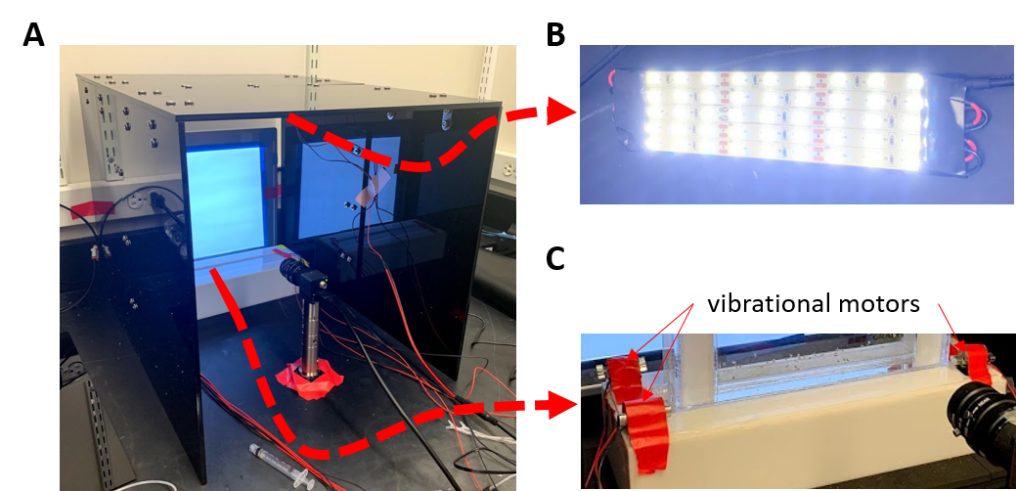


**Supplementary Figure 2.** Imaging setup

A) The backlight panel is placed behind the tank, the camera is positioned at the front of the tank, B) the white LED light strip is on top of the tank (underneath the black housing box) for phototaxis assay. C) The slot bracket allows for the placing of a tank at the exact same position for each imaging session and four vibrational motors are attached at the corner of the tank holder to generate controlled vibrational stimuli.


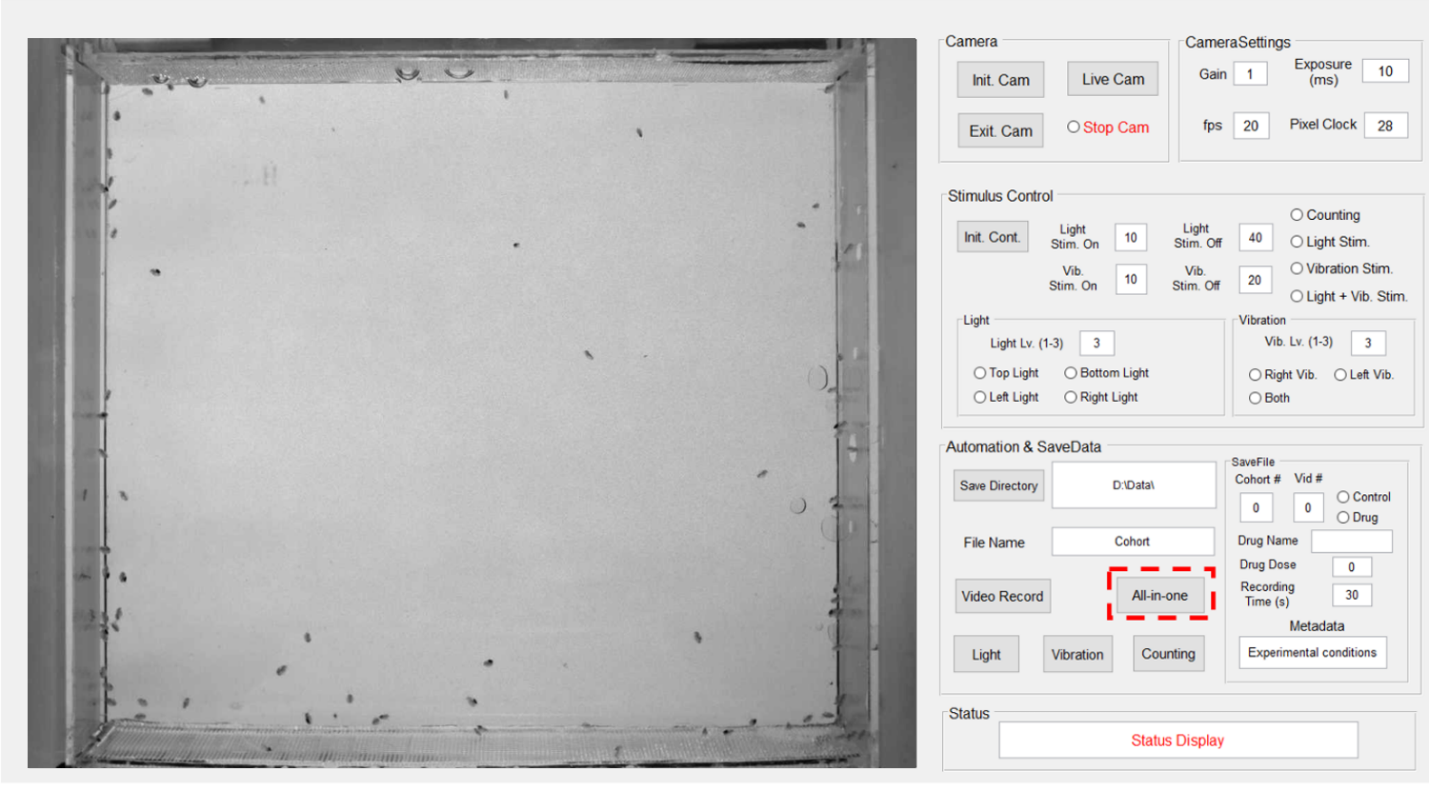


**Supplementary Figure 3.** The interface of the user GUI

The left window shows the currently monitored field of view. Right panels allow experimenters to manually control camera and stimulus (duration and intensity of both light and mechanical stimuli), save files with typed metadata, and view/take records of a tank in real-time. All these steps can be scripted and automatically performed by clicking an ‘All-in-one’ button. The current GUI status is shown at the bottom of the status panel.

The current recording routine consists of: acclimation (30 s); natural movement phase (30 s); single light stimulus (40 s); acclimation (20 s); multiple stimuli including weak/strong light and vibration (2 min). The recording time per tank is 4 min. Thus, the total time for 8 tanks is under 1 hour.


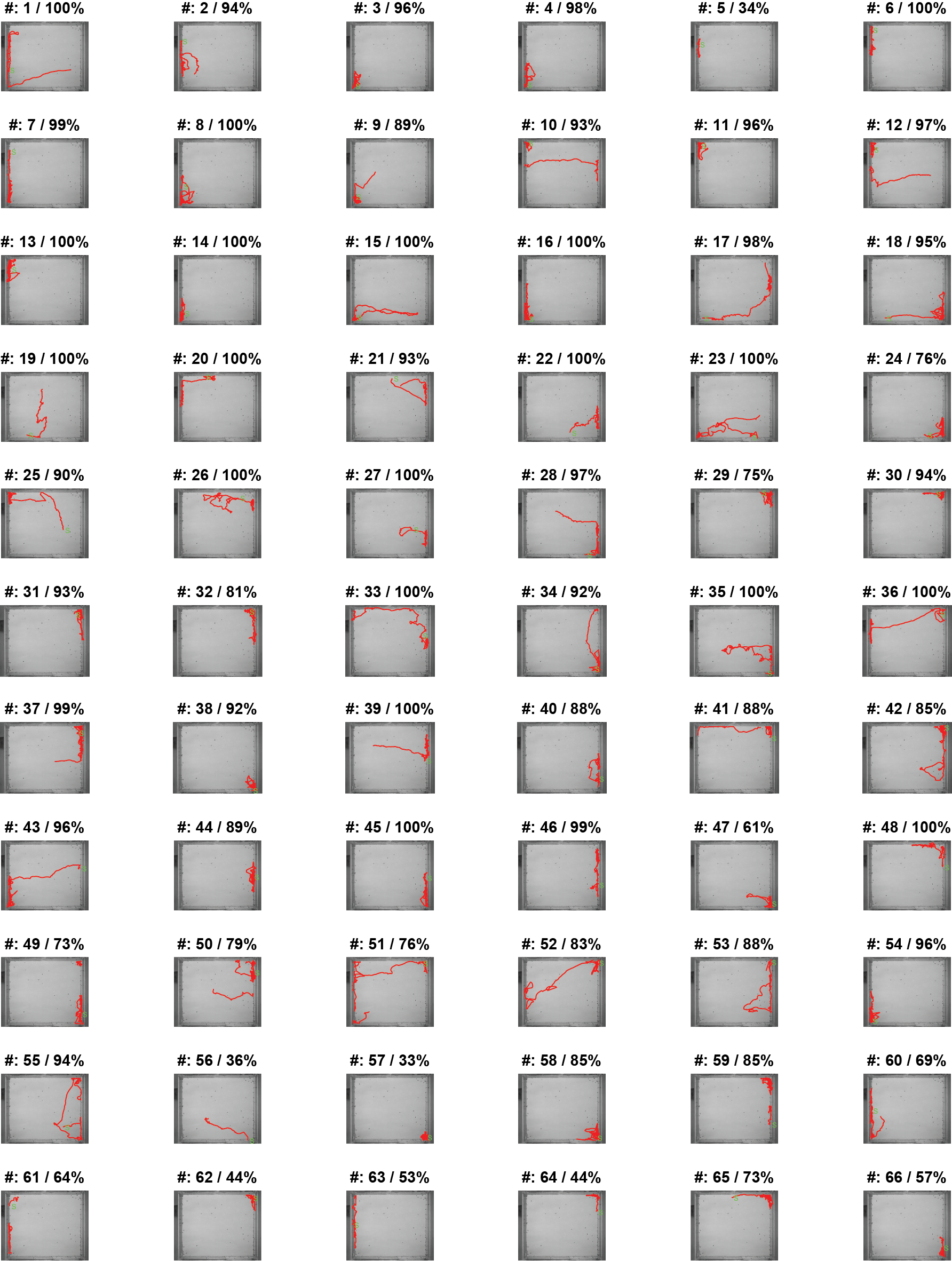


**Supplementary Figure 4**. Example trajectories from a video

Individual trajectories from Day 14 control 1 min long video (78 live animals). The red line indicates the automatically tracked trajectory of the animal. The green letter ‘S’ indicates the starting point. The percentage is showing how many frames are tracked. We extract behavioral parameters from each trajectory that got tracked during more than 20 s (33 %) in the 1 min video.

**
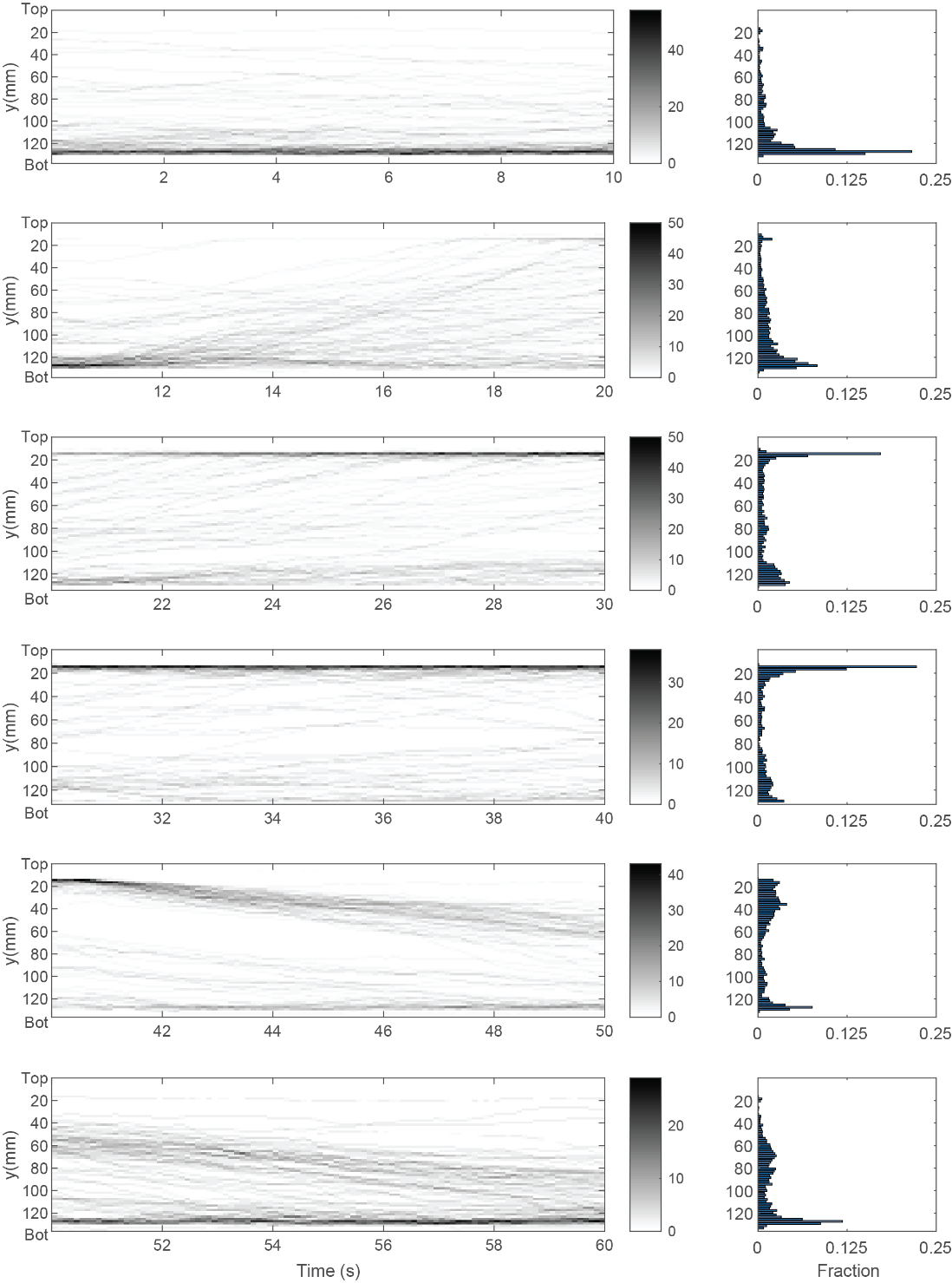
**

**Supplementary Figure 5.** Vertical movement of daphnids in the phototaxis experiment.

During this 1 min experiment, the light gets turned on at 10 s and then off at 40 s. Plots on the left show the vertical density of animals in the tank through time. Plots on the right show the histogram of animals’ location. In the beginning, most animals stay at the bottom of the tank. When the light is on (at 10 s), most animals start moving up towards it and remain at the top of the tank. Once the light is off (at 40 s), animals start returning to the bottom of the tank.

**
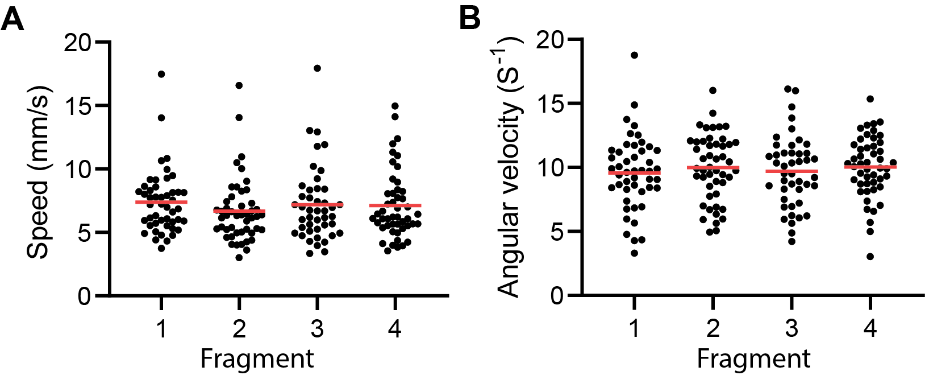
**

**Supplementary Figure 6.** The evidence of the measurement consistency

Comparison of speed and angular velocity across the 30 s long fragments of 1 min long video. All repeated data are not significantly different from one another. ANOVA test.


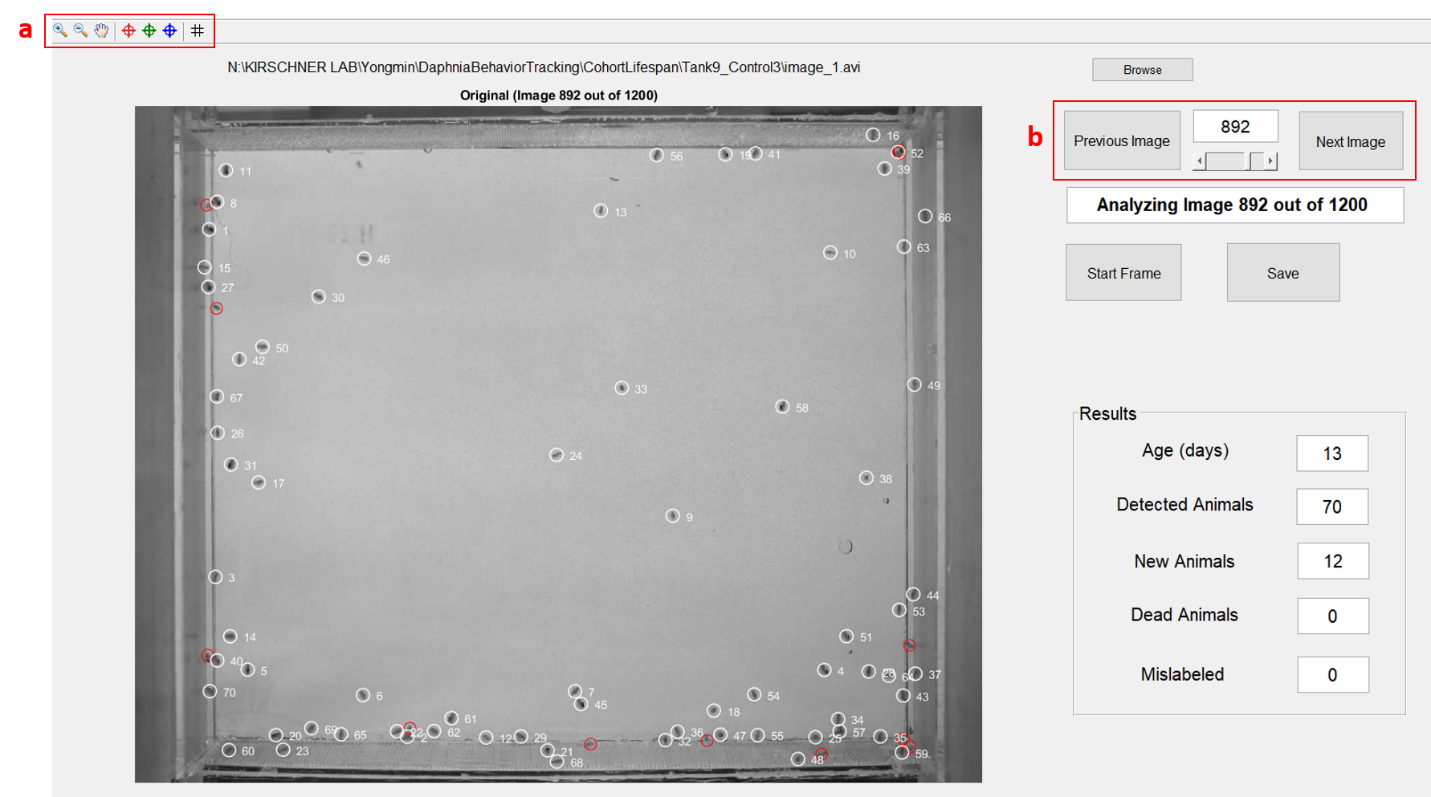


**Supplementary Figure 7.** MATLAB GUI for animal counting

After loading a video, the algorithm found a frame that might have the most identified animals and show the number of detected animals (labeled as a white circle and the ID number in the image) in the result panel. Using options in section a (red box), users can zoom-in / -out the image and add undetected animals (labeled as a red circle in the image), dead, or mislabeled animals. To change the frame, users can use the options in section b (red box); moving a frame by clicking the previous image or next image button or jumping ten frames by using a slide.

**
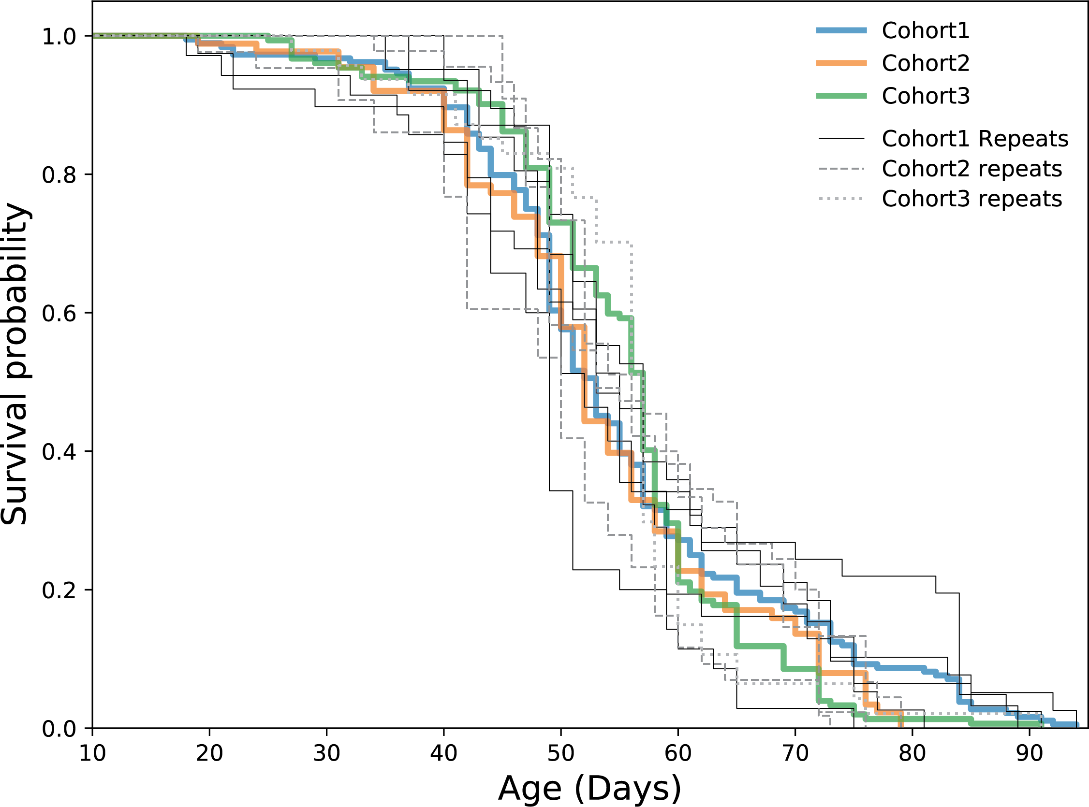
A**

**B**

**
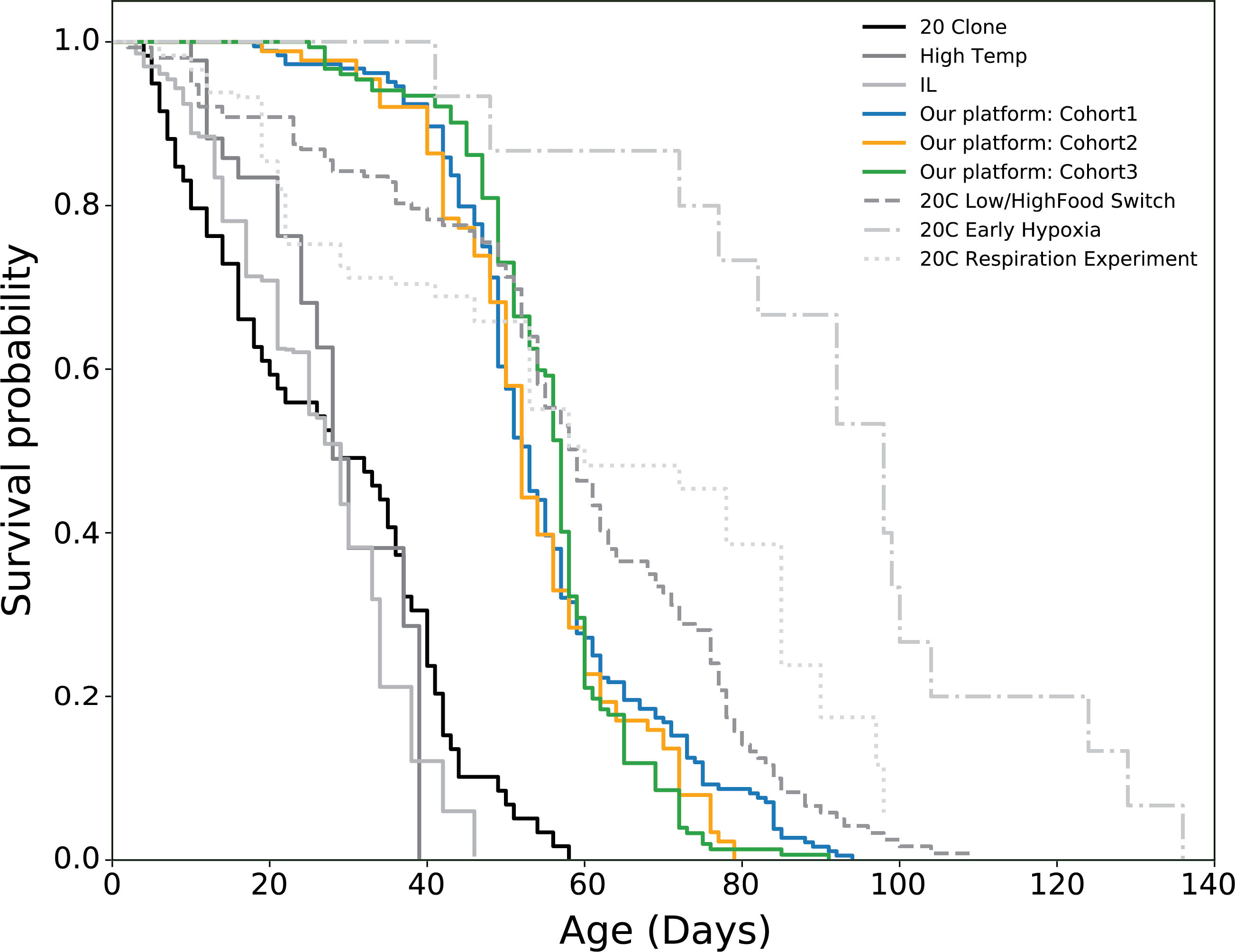
**

**C**

| **List of experiments** | | | | **Parametric survival fit** | | | | |
| --- | --- | --- | --- | --- | --- | --- | --- | --- |
| **Experiments** | **Cohort Size** | **Format** | **Temp (**$\boldsymbol{℃}$**)** | **Best (A)** | **Log-likelihood** | **Second best (B)** | **Log-likelihood** | **Significance (A vs B)** |
| 8 clones survey, IL data only* | 56 | 5 in 100 ml | 25 | Gen Gamma | -116.94 | Gompertz | -123.95 | Yes  ($\alpha$ < 0.001) |
| 20 clones survey(Coggins, Pearson, & Yampolsky, 2021) | 59 | 5 in 100 ml | 25 | Gen Gamma | -223.34 | Gompertz | -233.41 | Yes  ($\alpha$ < 0.001) |
| IL clone in High vs. Low food, High food data only* | 1201 | 5 in 100 ml | 25 | Gen Gamma | -3713.5 | Gompertz | -3729.2 | Yes  ($\alpha$ < 0.001) |
| 8 clones in High vs. Low food, high food and IL data only* | 152 | 5 in 100 ml | 20 | Gompertz | -623.27 | Gen Gamma | -625.77 | No  ($\alpha$ > 0.05) |
| 4 clones age-specific respiration, IL data only(Anderson et al., 2021) | 178 | 5 in 100 ml | 20 | Gen Gamma | -557.80 | Gompertz | -563.21 | Yes  ($\alpha$ < 0.005) |
| Early hypoxia, IL data only* | 15 | 1 in 20 ml | 20 | Weibull | -69.78 | Gompertz | -69.98 | No  ($\alpha$ > 0.05) |
| **This study** | | | | | | | | |
| Cohort 1 | 179 | "smart tanks" | 25 | Log  Logistic | -747.08 | Gen Gamma | -750.24 | Yes  ($\alpha$ < 0.05) |
| Cohort 2 | 86 |  | 25 | Gen Gamma | -347.15 | Weibull | -347.83 | No  ($\alpha$ > 0.05) |
| Cohort 3 | 149 |  | 25 | Gen Gamma | -577.12 | Weibull | -578.82 | No  ($\alpha$ > 0.05) |
| This study combined data |  |  | 25 | Log  Logistic | -1681.0 | Gen Gamma | -1684.1 | Yes  ($\alpha$ < 0.05) |

**Supplementary Figure 8.**

A) All survival curves (control condition). The survival curves set displays the total range of observed lifespan for each of the 10 experimental replicates (think black lines) from three cohorts at the control condition. Thick color lines show the averaged longevity of each cohort. Overall, the average longevity across the cohort experiment did not differ (log-rank test). We collect each cohort at different times (Collected date - Cohort 1: 10/14/2020; Cohort 2: 10/27/2020; Cohort 3: 11/11/2020). Cohort 1 - repeat 1: n = 38, repeat 2: n = 41, repeat 3: n = 35, repeat 4: n = 39, repeat 5: n = 31; Cohort 2 – repeat 1: n = 45, repeat 2: n = 43; Cohort 3 - repeat 1: n = 55, repeat 2: n = 47, repeat 3: n = 50). B) Comparison of survival curves from our platform (colored, same as on A) with survival curves obtained using “traditional” protocols (black or gray lines) in comparable conditions. Gray solid lines: three separate lifetable experiments with cohorts maintained at 25 ^o^C, gray dashed lines: three separate lifetable experiments with cohorts maintained at 20 ^o^C. “Traditional” protocol implied maintaining cohorts either with 5 individuals per 100 mL jar, or 1 individual per 20 mL vial with manual water change every 3-4 days. C) Results of parametric lifespan distribution fitting analysis using EM (Expectation-maximization) algorithm in both our platform (3 cohorts and combined data) and ‘traditional’ protocol-based experiments. Generalized Gamma (Gen Gamma), Weibull, Gamma, Exponential, Log-logistic (Log-log), Log-normal and Gompertz distributions fitted, the best and second-best fit reported. (*: unpublished data).


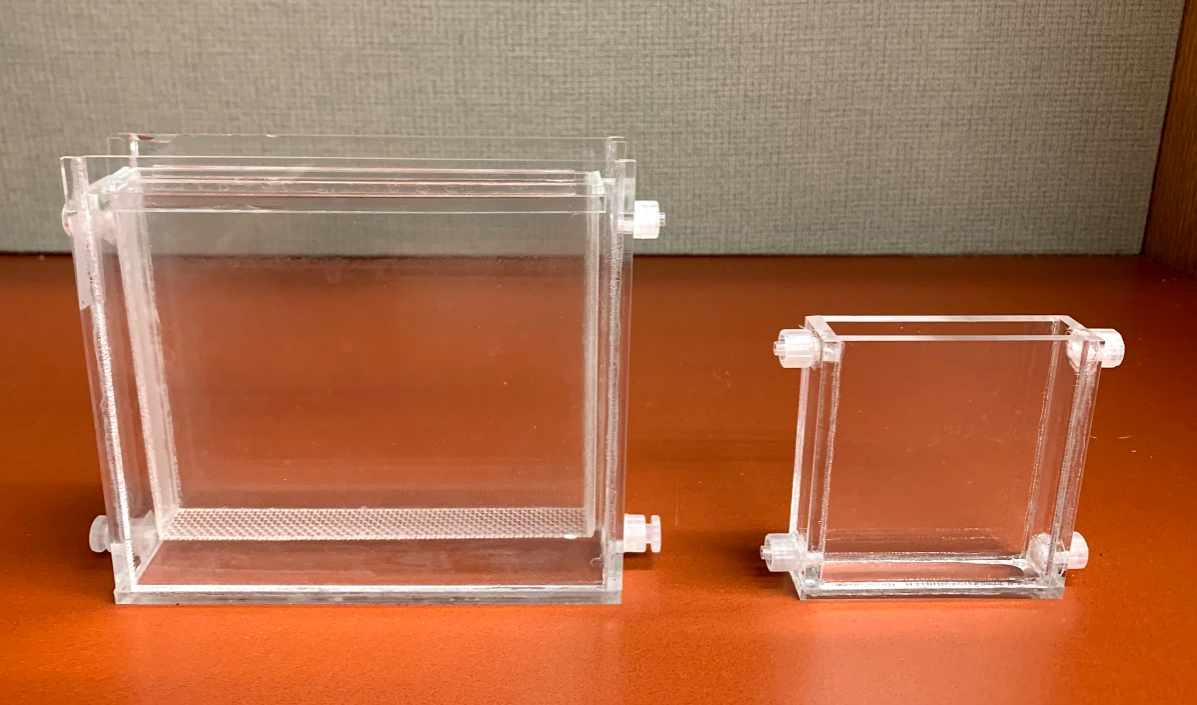


**Supplementary Figure 9.** Smaller tanks for short-term drug tests

Picture of the tanks for short-term drug tests. The volume of the left one is 400 ml (15 cm (w) x 13.5 cm (h) x 3.5cm (d)) and the right one is 100 ml (7.5 cm (w) x 8.5 cm (h) x 2 cm (d)).

**Supplementary Table 1**. A list and description of quantitatively extracted features

| **Experimental conditions** | **Category** | **Feature** | **Description** | **# of features** |
| --- | --- | --- | --- | --- |
| Both natural- and stimulus-swimming conditions | Swimming ability | Speed (SpdTop30, SpdBot30) | SpdTop30 (or SpdBot30) is consist of the fast  (or slow) 30^th^ percentile speed.  + standard deviation of speed | 4 |
|  |  | Swimming distance  for 10s | + Standard deviation | 2 |
|  |  | Angular velocity | + Standard deviation | 2 |
|  | Morphology | Size of transverse plane |  | 1 |
|  |  | Size of sagittal plane |  | 1 |
|  |  | Major axis | Major / minor axis of sagittal plane | 1 |
|  |  | Minor axis |  | 1 |
|  | Location in a tank | YFraction_Bot/Cen/Top | How long daphnids stay in each location. Equally divide a tank into 3 sections vertically | 3 |
|  | Descriptive features | forward fast running (FwdRun), forward swimming (Fwd), 'Forward slow swimming (FwdSlow), turning (Turn), spinning (Spin), and pause | FwdRun: 50% faster than total average speed (8.25 mm/s)  FwdSkiwL 50% slower than total average speed (2.75 mm/s)  Turn: Trajectory’s curvature is larger than 0.25  Spin: keep turn for 5 s | 6 |
| Only for stimulus condition | Swimming ability | Spd_1, 2, 3, 4, 5 and 6 | Calculate speed using a 10s-time window for each stimulus  1: before weak light stimulus  2: after weak light stimulus  3: before strong light stimulus  4: after strong light stimulus  5: before vibrational stimulus  6: after vibrational stimulus | 6 |
|  |  | VelY_1, 2, 3, 4, 5 and 6 | Calculate y-directional velocity (Vertical movement) using a 10s-time window for each stimulus  (condition is same as Spd) | 6 |

* Standard deviation: We calculated the standard deviation of measured behavioral parameters of individual animal’s swimming ability (not between individuals).

**Supplementary Table 2.** Results of using a simple linear regression to calculate adjusted R-squared, p-value, and mean squared error (RMSE) for individual features as a function of chronological age based on the data on natural swimming in control animals. We performed a linear regression on each feature for age with a training dataset and evaluated its accuracy on a test dataset (data were randomly divided 7:3 as train and test set).

| **Feature** | **Adjusted** $\boldsymbol{R}^{\boldsymbol{2}}$ | **RMSE** |
| --- | --- | --- |
| Major axis | 0.42 | 11.47 |
| Body size (Sagittal plan) | 0.36 | 11.97 |
| SD of Speed | 0.22 | 13.26 |
| Speed | 0.17 | 13.69 |
| SpeedTop30 | 0.20 | 13.39 |
| SD of distance | 0.16 | 13.70 |
| FwdRun | 0.16 | 13.75 |
| Minor axis | 0.16 | 13.75 |
| Distance for 10s | 0.12 | 14.04 |
| Pause | 0.06 | 14.48 |
| FwdSlow | 0.06 | 14.50 |
| SpeedBot30 | 0.05 | 14.63 |
| SD of Angular velocity | 0.04 | 14.69 |
| Body size (Transverse plan) | 0.03 | 14.71 |
| Angular velocity | 0.02 | 14.84 |
| YFractionCenter | 0.01 | 14.91 |
| Intensity | 0.01 | 14.93 |
| Turn | 0.00 | 14.96 |
| YFractionBottom | 0.00 | 14.94 |
| YFractionTop | 0.00 | 14.98 |
| Fwd | 0.00 | 14.96 |
| Spin | 0.00 | 15.07 |

**Supplementary Table 3**. Model prediction accuracy on test and testing data set

1. Training Data set

|  | **Natural Swimming (No stimulus)** | | **Phototactic responses (Light stimulus)** | |
| --- | --- | --- | --- | --- |
| **Model** | Adjusted $R^{2}$ | RMSE | Adjusted $R^{2}$ | RMSE |
| LASSO | 0.571 | 9.627 | 0.617 | 10.709 |
| Elastic Net | 0.577 | 9.566 | 0.616 | 10.709 |
| Random Forest | 0.634 | 6.369 | 0.866 | 6.371 |
| Gradient Boost | **0.865** | **2.018** | **0.942** | **3.821** |
| SVM | 0.570 | 9.662 | 0.613 | 10.858 |

2. Test Data set

|  | **Natural Swimming (No stimulus)** | | **Phototactic responses (Light stimulus)** | |
| --- | --- | --- | --- | --- |
| **Model** | Adjusted $R^{2}$ | RMSE | Adjusted $R^{2}$ | RMSE |
| LASSO | 0.547 | 9.669 | 0.634 | 10.472 |
| Elastic Net | 0.547 | 9.940 | 0.634 | 10.472 |
| Random Forest | 0.654 | 8.429 | 0.838 | 6.982 |
| Gradient Boost | **0.672** | **8.200** | **0.892** | **5.730** |
| SVM | 0.538 | 10.083 | 0.631 | 10.568 |

**Supplementary Table 4.** Calculated slope from the simple linear regression (forced x- and y-intercepts are zero).

| **Condition** | **Natural** | **Stimulus** |
| --- | --- | --- |
| Control | 0.9639 $\pm$ 0.0005 | 0.9833 $\pm$ 0.0007 |
| Metformin 1 $\mu M$ | 1.0410 $\pm$ 0.0049 | 1.1600 $\pm$ 0.0036 |
| Metformin 0.1 $\mu M$ | 0.9280 $\pm$0.0042 | 0.9264 $\pm$ 0.0030 |
| Metformin 0.01 $\mu M$ | 0.8228 $\pm$ 0.0040 | 0.9450 $\pm$ 0.0042 |

**Supplementary Note 1 – Platform design documentation**

The following documentation is a overview on how the entire platform was built and operated for the set of experiments shown in the article. By design, our system is modular and scalable. As a result, it can be customized depending on the user’s requirements.

**1.1. Tank design**

Dimensions for the tank (A: Housing tank, B: Cap and C: Insert). All dimensions are given in millimeters. Sections through the device in front, bottom and side views are shown.

**
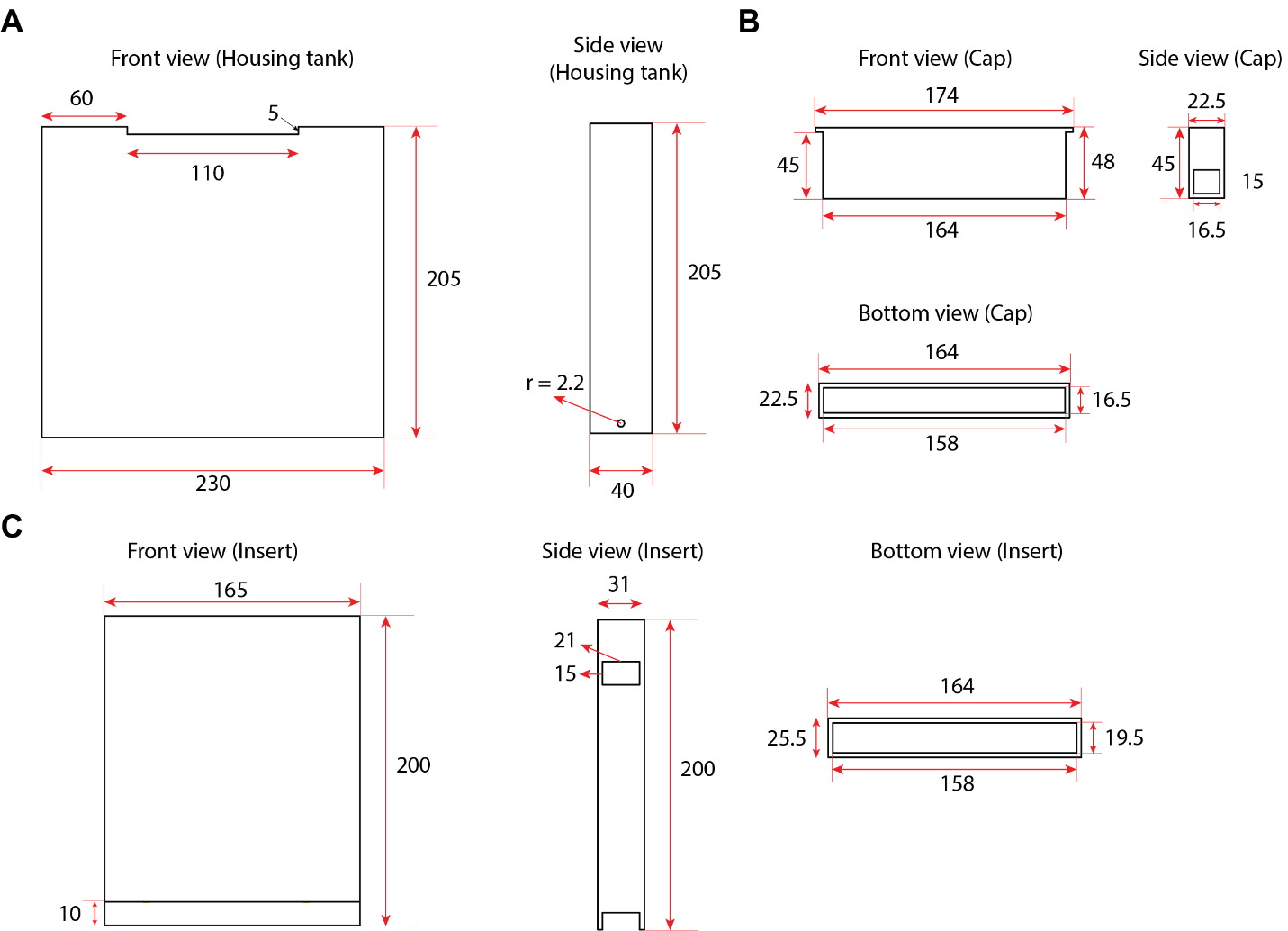
**

**1.1.1. The advantage of 2.5D tank design**

The dimension of the insert tank is 2.5D (e.g., the size of the swimming arena is 16.5 cm (w) x 14.5 cm (h) x 2.5 cm (d)) which means a large xy area and short z-dimension. In addition, we used very weak background light to create even illumination that helps to make animals align the backside of the tank (*Daphnia magna* exhibits positive phototactic behaviors). In this circumstance, animals usually move through the xy dimension instead of moving through the z-direction (see movie 3). Thus, a single camera is good enough to appropriately capture various behavioral and morphological features.

**1.2. Platform part list**

| **Category** | **Subcategory** | **Item name / Description** | **Vendor** | **Part number** | **Quantity** | **Unit Price ($)** | **Note** |
| --- | --- | --- | --- | --- | --- | --- | --- |
| **Tank system** | Tank | Clear Cast Acrylic Sheet, 12" x 24" x 3/16" | McMaster-Carr | 8560K219 | Depending on the number of tank | 22.63 | For housing tank |
|  |  | Clear Cast Acrylic Sheet, 12" x 24" x 1/16" | McMaster-Carr | 8560K172 |  | 10.09 | For insert and cap |
|  |  | Cast Acrylic Sheet, 12" x 24" x 1/8", Color: White | McMaster-Carr | 8505K742 |  | 15.04 | For the partition |
|  |  | SCIGRIP 16 Acrylic Cement | Amazon | B005ZH31W2 | 1 | 28.65 |  |
|  |  | Female Luer Thread Style Cap | FTLLP-6005 | Nordson Medical | Pack of 100 | 18 | For outlet of the tank |
|  |  | Male Luer Integral Lock | XMTLL-6005 | Nordson Medical | Pack of 100 | 38 |  |
|  | Air control | Air Stones | Amazon | B076S3D75C | Pack of 12 | 5.99 |  |
|  |  | Air Filter | McMaster-Carr | 8395K11 | 1 | 66.17 |  |
|  |  | Air tubing | McMaster-Carr | 5548K82 | 100ft | 34 |  |
|  |  | Compact Compressed Air Regulator | McMaster-Carr | 9892K11 | 1 | 39.74 |  |
| **Imaging system** | Imaging | Camera / High-Sensitivity USB 3.0 CMOS Camera | Thorlabs | DCC3240M | 1 | 1,336.73 |  |
|  |  | 8 mm EFL, f/1.4, for 2/3" C-Mount Format Cameras | Thorlabs | MVL8M23 | 1 | 238.49 |  |
|  |  | Rosco Roscolux 398 Neutral Grey | stagelightingstore | Rosco Roscolux Sheet R398 | 1 | 6.99 | ND filter |
|  |  | Artograph LightPad, | Amazon | B003N45KRS | 1 | 139.99 | 12 x 12 inch |
|  | Control & Automation | Arduino uno | Arduino | ARDUINO UNOREV3 | 1 | 19.55 |  |
|  |  | LED Light Strip | Amazon | B075RYSHQQ | 1 | 14.99 |  |
|  |  | Vibration Motor | Amazon | B07RGX3R1Q | Pack of 5 | 13.99 | Vibrational stimulus |
|  |  | Acoustic Damper | Amazon | B01M1EQHDO | 1 | 15.99 | Put the underneath of tank holder |

**1.3. Cost of the platform**

The total cost to run 8 tanks for ~400 individuals is ~$2,230. Importantly, the fabrication cost for tanks is low (~$212 is required to fabricate 8 tanks), thus the platform is easily scalable.

**Supplementary Note 2 – Phenotype tracking algorithm**

**2.1. Overview**

Daphnia phenotype tracking was performed using a multistep process beginning with raw video data. All analyses were performed in MATLAB 2020a using custom-written scripts, based in part on the previous algorithms(Albrecht & Bargmann, 2011; Le et al., 2020) to segment video frames and identify continuous centroid paths of individual daphnids. Each frame was turned into a binary image using adaptive background correction and thresholding method. The resulting binary image was analyzed using a consensus approach informed by positional and size parameters to separate the target objects (adult Daphnia) from other objects and then performed tracking followed by measurements of the morphological and behavioral parameters.

**2.2. The workflow of the algorithm**

1) Setup parameters depending on the experimental conditions in the *DaphniaPhenotyping* (Main function)*.*

2) Background subtraction and object segmentation and detection in the *DaphniaTracker.*

*3)* Extract various phenotypic parameters in the *DaphniaSegmentTracks.* Users can tune the feature extraction setting parameter in the *InitialSettings*.

4) Visualize the extracted features via *Ethogram* and *DaphniaDensity*.

**2.3.** **Validation of the automated *Daphnia* detection algorithm**

To validate the accuracy of the *Daphnia* detection algorithm, we used a video that has 17 alive animals. Over 100 frames, 1,700 individual Daphnia could be detected. 92.88% of individuals were accurately detected (e.g., We manually checked the centroid of detected objects).

**Supplementary Note 3 – Live-dead assay**

As *Daphnia* died in the tank, they stopped the movement and their body turned to be more transparent. Then they were decomposed and disappeared within 2 days (e.g., not detectable by eye and camera), which makes it easy to conduct live-dead assays. We excluded dead animals when it does not move through the entire recorded time. Specifically, our automated tracking algorithm does not identify dead animals using the background subtraction method. However, to avoid any counting error, we checked all counted data manually and corrected it. The accuracy of automated counting is vary depending on the density of animal populations (>40 animals: 71% accuracy; <20 animals: 81% accuracy). The error rate is 0.29% that includes detecting non-daphnia and dead animals.

**3.1. Example image of a dead animal**


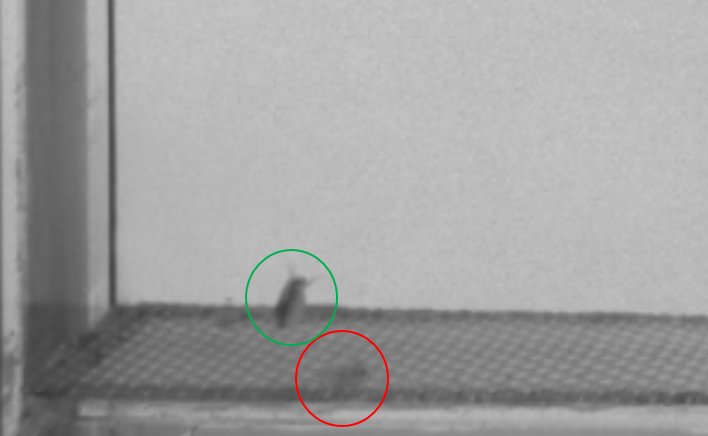


A comparison of a live animal (green circle) to an almost transparent body of an animal which died a day ago. In one day the dead body almost completely decomposes and disappeares.

**Supplementary Note 4 – Comparison median lifespan**

**4.1. List of *Daphnia magna’s* median lifespan from literature**

| Median Lifespan | Temp ($℃$) | Sample Size | Water | Light | References |
| --- | --- | --- | --- | --- | --- |
| 54 | 25 | 424 | ADaM | 16L:8D | This study |
| 26 | 20 | 10 | Aged tap water | 12L:12D | Schwarzenberger et al, 2014(Schwarzenberger, Christjani, & Wacker, 2014) |
| 33 | 20 | 20 | Aged tap water | 16L:8D | Schwarzenberger et al, 2014(Schwarzenberger et al., 2014) |
| 40 | 20 | 30 | Conditioned lake water | 16L:8D | Pietrzak et al, 2010(Pietrzak, Grzesiuk, & Bednarska, 2010) |
| 48 | 20 | 30 | Conditioned lake water | 16L:8D | Pietrzak et al, 2010(Pietrzak et al., 2010) |
| 56 | 20 | 30 | Conditioned lake water | 16L:8D | Pietrzak et al, 2010(Pietrzak et al., 2010) |
| 56 | 20 | 40 | Conditioned lake water | 15L:9D | Pietrzak et al, 2020(Pietrzak et al., 2010) |
| 57 | 20 | 30 | Conditioned lake water | 16L:8D | Pietrzak et al, 2010(Pietrzak et al., 2010) |
| 57 | 23 | 14 | ADaM |  | Nguyen et al, 2021(Nguyen, Matsuura, Kato, & Watanabe, 2021) |
| 68 | 19 |  | Aged tap water | 16L:8D | Korpelainen et al, 1986(Korpelainen, 1986) |
| 52 | 24 |  | Aged tap water | 16L:8D | Korpelainen et al, 1986(Korpelainen, 1986) |
| 31 | 29 |  | Aged tap water | 16L:8D | Korpelainen et al, 1986(Korpelainen, 1986) |
| 82 | 20 | 30 |  | 16L:8D | Barbosa et al, 2017(Barbosa, Inocentes, Soares, & Oliveira, 2017) |
| 83 | 20 | 50 |  | 16L:8D | Constantinou et al, 2019(Constantinou, Sullivan, & Mirbahai, 2019) |
| 87 | 21 | 80 |  | 16L:8D | Orlansky et al, 2019(Orlansky & Ben-Ami, 2019) |
| 105 | 21 | 10 | ADaM | Permenent light | Menzel et al, 2008(Euent, Menzel, & Steinberg, 2008) |
| 64 | 18 |  | Aerated tap water | 8L:16D | Kelpsiene et al, 2020(Kelpsiene, Torstensson, Ekvall, Hansson, & Cedervall, 2020) |
| 45 | 21 | 14 |  | 14L:10D | Seyoum et al, 2020(Seyoum, Pradhan, Jass, & Olsson, 2020) |
| 62 | 19 | 20 |  | 14L:10D | Martinez-Jeronimo et al, 1994(Martinez-Jeronimo, Villaseñor, Rios, & Espinosa, 1994) |
| 95 | 20 | 751 |  | 16L:8D | Lohr et al, 2014(Lohr, David, & Haag, 2014) |
| 80 | 25 | 563 |  | 16L:8D | Lohr et al, 2014(Lohr et al., 2014) |

**4.2. Plot the median lifespan across various literature (data from above Table 4.1).**

**Supplementary Note 5 – Comparison operation time between lifetable experiment and our platform**

Unit: hours/day

Note:

- Setup Time: Preparing arrays of jars (manual assay) or tanks (our platform), including arranging, labeling, filling with medium

1) Manual assay: 0.25 min / container

2) Our platform: 6 min/tank (includes moving the tank from an incubator to an imaging setup and vice versa; experimenter can change the water (~1 min/tank) while another tank is in an automated imaging session).

- Handling Time: Handling animals during water changing and counting.

For our platform format, we assume to conduct recording three videos for counting and monitoring behaviors in various situations including natural movement, single light stimulus and combination of light and vibration stimulus as this study did.

1) Manual assay: 0.5 min / animal

2) Our platform: No individual animal handling is required.

- We assume that experimenters will count animals every 4^th^ day. Thus, a session of the cohort experiment is staggered over three days.

- For the manual assay, around 2,000 cohort size is the maximum for one researcher (e.g., require > 8 hours per day for the ‘Manual 1 animal in 20 ml tube’ experiment ; >6 hours per day for the ‘Manual 5 animals in 100 ml jar’ experiment). However, one researcher can handle more than 100,000 animals using our platform (e.f.g, require < 7 hours per day). Furthermore, if multiple experiments participate in the manual assay, it is easier to make errors including animal handling, counting, recording keeping, etc. On the other hand, our platform highly reduces the experimental bias because it does not require individual animal handling.

**Supplementary Video 1.** Video shows how neonates (n = 34, day 1 - 3) are separated through mesh and continuous flow in the tank. Separated neonates are captured at the cap (top small size mesh). 10x playback.

**Supplementary Video 2.** Animals’ responses to 30s of light stimulus between 10th and 40^th^ seconds in a 1 min recording. The top right plot shows the relative location of individual animals across recoding time. The bottom right plot shows the average speed of animals. When light is turned on or off, animals show substantial acceleration. 5x playback.

**Supplementary Video 3.** Video shows how the algorithm tracks the centroid and detects the body size of the animal. Real-time playback.

**Supplementary Video 4.** Video shows animals’ natural swimming in 3% ethanol condition. The left panel was recorded between 0 ~ 1min and the right one was recorded between 4~5min. Each color represents an individual’s trajectory. 5x playback.

**References**

Albrecht, D. R., & Bargmann, C. I. (2011). High-content behavioral analysis of Caenorhabditis elegans in precise spatiotemporal chemical environments. *Nature methods, 8*(7), 599.

Anderson, C. E., Ekwudo, M. N., Jonas-Closs, R. A., Cho, Y., Peshkin, L. M., Kirschner, M. W., & Yampolsky, L. Y. (2021). Lack of Age-related Respiratory Changes in Daphnia. *bioRxiv*.

Barbosa, M., Inocentes, N., Soares, A. M., & Oliveira, M. (2017). Synergy effects of fluoxetine and variability in temperature lead to proportionally greater fitness costs in Daphnia: a multigenerational test. *Aquatic Toxicology, 193*, 268-275.

Coggins, B., Pearson, A., & Yampolsky, L. (2021). Does geographic variation in thermal tolerance in Daphnia represent trade-offs or conditional neutrality? *Journal of Thermal Biology, 98*, 102934.

Constantinou, J., Sullivan, J., & Mirbahai, L. (2019). Ageing differently: Sex-dependent ageing rates in Daphnia magna. *Experimental gerontology, 121*, 33-45.

Euent, S., Menzel, R., & Steinberg, C. E. (2008). GENDER-SPECIFIC LIFESPAN MODULATION IN DAPHNIA MAGNA BY A DISSOLVED HUMIC SUBSTANCES PREPARATION. *Annals of Environmental Science*.

Kelpsiene, E., Torstensson, O., Ekvall, M. T., Hansson, L. A., & Cedervall, T. (2020). Long-term exposure to nanoplastics reduces life-time in Daphnia magna. *Sci Rep, 10*(1), 5979. doi:10.1038/s41598-020-63028-1

Korpelainen, H. (1986). The effects of temperature and photoperiod on life history parameters of Daphnia magna (Crustacea: Cladocera). *Freshwater Biology, 16*(5), 615-620.

Le, K. N., Zhan, M., Cho, Y., Wan, J., Patel, D. S., & Lu, H. (2020). An automated platform to monitor long-term behavior and healthspan in Caenorhabditis elegans under precise environmental control. *Communications Biology, 3*(1). doi:10.1038/s42003-020-1013-2

Lohr, J. N., David, P., & Haag, C. R. (2014). Reduced lifespan and increased ageing driven by genetic drift in small populations. *Evolution, 68*(9), 2494-2508.

Martinez-Jeronimo, F., Villaseñor, R., Rios, G., & Espinosa, F. (1994). Effect of food type and concentration on the survival, longevity, and reproduction of Daphnia magna. *Hydrobiologia, 287*(2), 207-214.

Nguyen, N. D., Matsuura, T., Kato, Y., & Watanabe, H. (2021). DNMT3.1 controls trade-offs between growth, reproduction, and life span under starved conditions in Daphnia magna. *Sci Rep, 11*(1), 7326. doi:10.1038/s41598-021-86578-4

Orlansky, S., & Ben-Ami, F. (2019). Genetic resistance and specificity in sister taxa of Daphnia: insights from the range of host susceptibilities. *Parasit Vectors, 12*(1), 545. doi:10.1186/s13071-019-3795-y

Pietrzak, B., Grzesiuk, M., & Bednarska, A. (2010). Food quantity shapes life history and survival strategies in Daphnia magna (Cladocera). *Hydrobiologia, 643*(1), 51-54.

Schwarzenberger, A., Christjani, M., & Wacker, A. (2014). Longevity of Daphnia and the attenuation of stress responses by melatonin. *BMC physiology, 14*(1), 1-7.

Seyoum, A., Pradhan, A., Jass, J., & Olsson, P. E. (2020). Perfluorinated alkyl substances impede growth, reproduction, lipid metabolism and lifespan in Daphnia magna. *Sci Total Environ, 737*, 139682. doi:10.1016/j.scitotenv.2020.139682
